# Chromosome-level genome assembly and single-cell analysis unveil molecular mechanisms of arm regeneration in the ophiuroid *Ophiura sarsii vadicola*

**DOI:** 10.1186/s13059-025-03542-5

**Published:** 2025-03-31

**Authors:** Qin-Zeng Xu, Yi-Xuan Li, Wen-Ge Shi, Yue Dong, Zhong Li, Jack Chi-Ho Ip, Matthew P. Galaska, Chen Han, Qian Zhang, Yu-Yao Sun, Lin-Lin Zhao, Kai-Ming Sun, Zong-Ling Wang, Jian-Wen Qiu, Xue-Lei Zhang

**Affiliations:** 1 MNR Key Laboratory of Marine Eco-Environmental Science and Technology, First Institute of Oceanography, Ministry of Natural Resources, Qingdao, PR China; 2 Laboratory for Marine Ecology and Environmental Science, Qingdao Marine Science and Technology Center, Qingdao, PR China; 3 Department of Biology, Hong Kong Baptist University, Hong Kong SAR, PR China; 4 Science Unit, Lingnan University, Hong Kong SAR, PR China; 5 Pacific Marine Environmental Lab, National Oceanic and Atmospheric Administration, Seattle, WA USA; 6 Anchor QEA, Seattle, WA, USA; 7 Institute of Oceanographic Instrumentation, Qilu University of Technology (Shandong Academy of Sciences), Qingdao, PR China

## Abstract

**Background:**

Ophiuroids, belonging to Ophiuroidea in Echinodermata, possess remarkable regenerative capacities in their arms, relying on cellular recruitment and de-differentiation. However, limited high-quality genomic resources have hindered the investigation of the underlying molecular mechanisms of ophiuroid regeneration.

**Results:**

Here, we report a chromosome-level genome of *Ophiura sarsii vadicola*, 259.28 Mbp in length with a scaffold N50 length of 66.91 Mbp. We then perform bulk and single-cell RNA sequencing analysis to investigate gene expression and cellular dynamics during arm regeneration. We identify five distinct cellular clusters involved in the arm regeneration and infer the dynamic transformations from sensory stimulation to injury response, wound healing, and tissue regeneration. We find that progenitor cells derived from connective tissue cells differentiate into muscle, cartilage, endothelial, and epithelial cells. Pseudotime analysis indicates that muscle differentiation occurs early in the regeneration process.

**Conclusions:**

Our genomic resource and single-cell atlas shed light on the mechanisms of organ regeneration in ophiuroids.

**Supplementary Information:**

The online version contains supplementary material available at 10.1186/s13059-025-03542-5.

## Background

Regeneration is a common and essential process across all organisms, from unicellular to multicellular forms, and occurs in both invertebrates and vertebrates, although regenerative capacities vary among different taxa [1, 2]. Echinodermata, a phylum of deuterostomes sharing a common ancestor with Chordata, possesses robust and rapid regenerative capacity [3]. Organ regeneration is a prevalent characteristic across all five classes of the phylum Echinodermata in response to trauma and occurs in various body parts, including arms [4–6], internal organs [7, 8], and even across the entire body [9]. Stellate echinoderms, including crinoids, asteroids, and ophiuroids, display unique combinations of regenerative abilities through re-epithelialization, inflammatory responses, and remodeling of post-injury tissues, with arm regeneration being one of the most extensively studied processes [10, 11].

Brittle stars (Ophiuroidea) are the most diverse group of echinoderms, comprising over 2100 species worldwide [12]. Ophiuroids have emerged as an ideal model for studying organ regeneration in both natural [13] and experimental settings [14, 15], due to their exceptional regenerative abilities and ease of laboratory maintenance. Their rapid regeneration rates [16], distinct morphogenesis [6], diverse morphologies [17], and flexible locomotion with different arm vertebrae shapes [18] provide valuable insights into regeneration mechanisms in deuterostomes and the evolution of regenerative capabilities across taxa.

Studies have been conducted to understand several aspects of arm regeneration in brittle stars [6, 19]. Arm regeneration comprises four main stages: wound healing/repair, early regeneration, intermediate regeneration, and advanced regeneration, each marked by specific cellular and molecular events [14]. Interactions between wound healing and early regeneration stages, coupled with the transmission of cytoplasmic molecular regeneration signals, determine whether ophiuroids successfully progress through all four stages [14]. In recent decades, several studies have sought to unveil the underlying gene regulatory networks involved in different regeneration phases, employing various molecular and cellular techniques, including in situ hybridization, immunohistochemistry [20], proteomics [15], and transcriptomics [21–23]. Notably, two prominent signaling pathways were found to play key roles during arm regeneration: the fibroblast growth factor (FGF) pathway [24], which activates fibroblasts and facilitates cellular proliferation and tissue repair, and the Wnt [24, 25] pathway, which stabilizes stem cells and promotes their differentiation. However, uncertainties remain regarding arm regeneration, including the comprehensive understanding of cellular dynamics, cell type interactions during the regenerative process, and the signaling pathways and gene regulatory networks involved in each cell type.

Over the past decade, the emergence of single-cell RNA sequencing (scRNA-seq) has revolutionized the study of regeneration mechanisms [26]. This powerful technique has provided new insights into cellular composition [27], gene expression dynamics, and cell differentiation trajectories during regeneration [28]. Moreover, scRNA-seq has revealed previously unknown cell types and identified unique gene expression signatures associated with specific regeneration stages [3]. This approach has been successfully applied to investigate regeneration in diverse model organisms, including earthworm segments [29, 30], mouse muscles [31], African clawed frog tails [32], and axolotl limbs [33]. These investigations highlight the potential of single-cell analysis in elucidating arm regeneration mechanisms in brittle stars. However, the limited availability of high-quality reference genomes for the class Ophiuroidea, with only two published genomes in 2024 [34, 35], has constrained the application of scRNA-seq in understanding the molecular mechanisms of regeneration in ophiuroids [16].

The brittle star *Ophiura sarsii vadicola* (Ophiurida: Ophiuridae) is a dominant epibenthos in the Yellow Sea [36, 37] that exhibits remarkable natural regeneration capacity. In this study, we generated a high-quality chromosome-level reference genome for *O. sarsii vadicola* to facilitate comparative genomic and transcriptomic analyses of its regeneration capacity. To gain insights into the molecular mechanisms underlying wound repair and early regeneration stages, we employed scRNA-seq technology to generate a dynamic multicellular transcriptomic profile spanning regeneration stages. By analyzing amputation tissues collected from different time points, we elucidated the composition and gene expression dynamics of cellular constituents involved in arm repair and regeneration. Leveraging the findings from single-cell analysis, we proposed a hypothesis regarding the transformation of cell types during different stages of arm regeneration, providing a foundation for future investigations into echinoderm regeneration mechanisms.

### Genome assembly and characterization

**Fig. 1:**
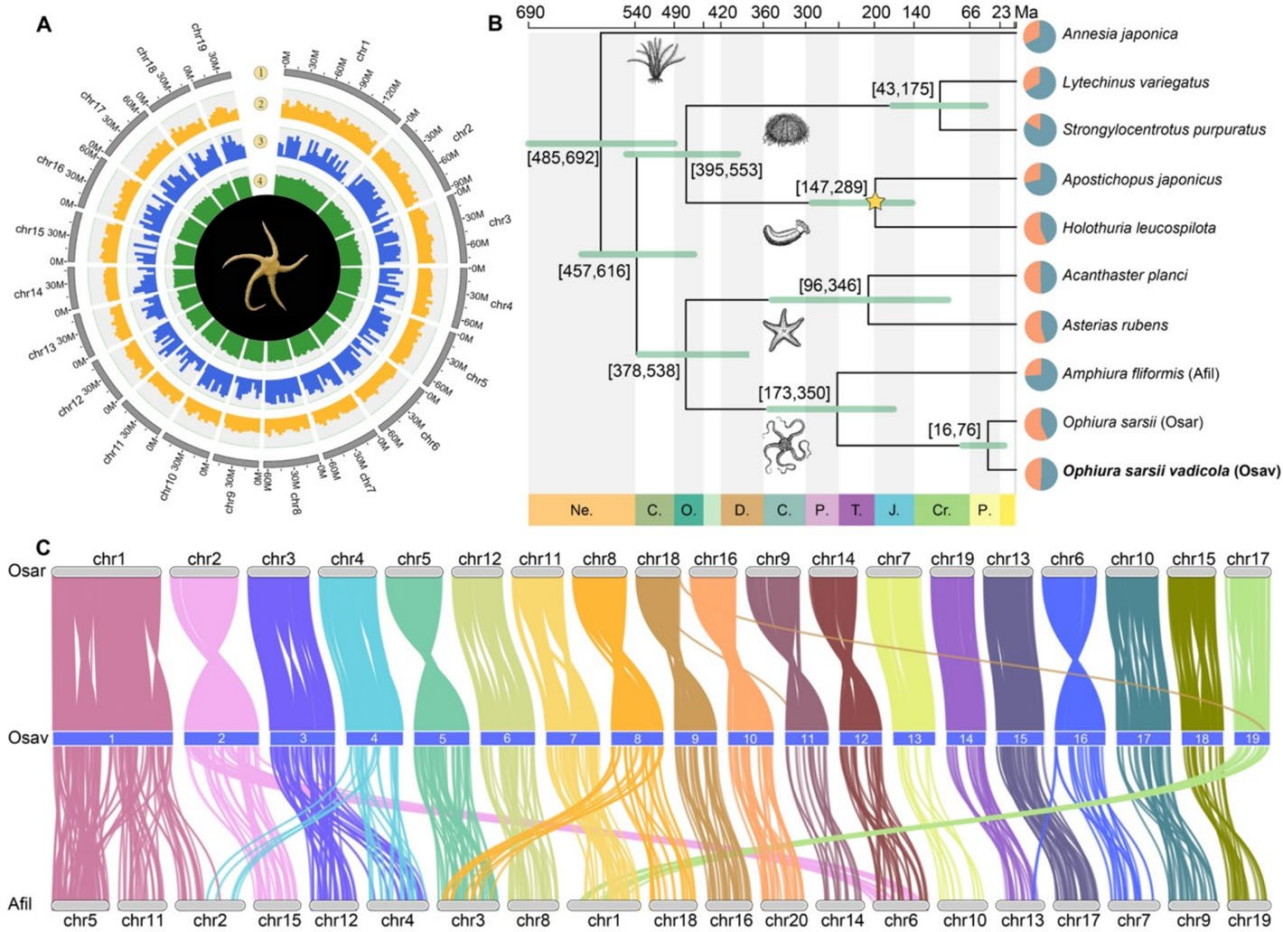
Genomic features of *Ophiura sarsii vadicola* and phylogenetic relationships among echinoderms with available genome data. **A** Circos plot of *Ophiura sarsii vadicola* genomic features with an image of an *O. sarsii vadicola* specimen (5 cm in size). Circles show (1) the length of each chromosome, (2) the density of genes, (3) the density of repetitive sequences, and (4) GC content. **B** Phylogenetic relationships and divergence times of ten echinoderms with genome resources. A yellow asterisk marks the node where time calibration was applied for divergence time analysis. The pie diagram (orange and blue) shows the percentage of expanded and contracted gene families for each tip species. **C** Collinearity relationships between *O. sarsii vadicola* (Osav) and *O. sarsii* (Osar), and between *O. sarsii vadicola* and *Amphiura filiformis* (Afil)

Specimens of *O. sarsii vadicola* were collected from the Yellow Sea for laboratory culture and genome sequencing. A total of 143.7 Gbp (114.47×) of PacBio SMRT long reads and 218.32 Gbp (173.92×) of Illumina short reads were generated for genome assembly (Additional file 2: Table S1). K-mer analysis estimated the size of *O. sarsii vadicola* genome to be 1.21 Gbp with a heterozygosity rate of 2.21% (K-mer = 17, Additional file 1: Fig. S1). Using a hybrid assembly approach with 362.02 Gbp of sequence data, we assembled a 1.27 Gbp genome, consisting of 1988 contigs with an N50 of 2.44 Mbp and GC content of 37.2% (Additional file 2: Table S2). After HI-C scaffolding, 99.62% of the assembled contigs were anchored into 19 chromosomes, yielding a final genome size of 1.27 Gbp across 216 scaffolds with an N50 of 66.91 Mbp (Fig. 1A, Additional file 1: Fig. S2 and Additional file 2: Tables S3–S4). Repeat sequences constituted 54.8% of the assembly (Additional file 2: Table S5), predominantly comprising long terminal repeats (LTRs, 48.69%).

We validated the genome assembly quality using multiple methods. The high quality of the genome assembly was demonstrated by the 96.62% mapping rate of Illumina reads covering 99.59% of the genome with an average sequencing depth of 143.30× (Additional file 2: Table S6). The quality value (QV) calculated with the Merqury package was 36.88, indicating 99.9% accuracy. Genome completeness, assessed using the Core Eukaryotic Genes Mapping Approach (CEGMA), showed 93.95% of the 248 core eukaryotic genes (Additional file 2: Table S7). The genome encodes 26,226 protein-coding genes, with an estimated 94.0% Benchmarking Universal Single-Copy Orthologs (BUSCO) completeness score; 92.3% of these were annotated (Additional file 1: Fig. S3 and Additional file 2: Table S7). The average transcript length was 22,018 bp (exons + introns), with mean lengths for coding sequences (CDS, 1583.07 bp), exons (218.82 bp), and introns (3277.68 bp) comparable to those in other echinoderms (Additional file 1: Fig. S4 and Additional file 2: Tables S8–S9).

A total of 10 echinoderm genome assemblies were used to conduct comparative analysis (Fig. 1B). We identified 1537 single-copy orthogroups among a total of 27,305 orthogroups. *O. sarsii vadicola* exhibited 48 significantly expanded gene families (Additional file 2: Table S10), including the coagulation factor 5/8 C-terminal domain and the discoidin domain (FA58C), both known to be involved in post-amputation coagulation. Phylogenetic analysis confirmed crinoids as the basal echinoderm lineage, with ophiuroids most closely related to asteroids, and echinoids grouped with holothuroids. The ophiuroid-asteroid and echinoid-holothuroid clades diverged around 378–553 Ma, with ophiuroid diversification occurring earlier than other clades (170–350 Ma).

The three brittle star species shared 11,588 orthogroups, with *O. sarsii vadicola*, *O. sarsii*, and *Amphiura filiformis* possessing 269, 381, and 1444 species-specific orthogroups respectively (Additional file 1: Fig. S5). Enrichment analysis of species-specific orthogroups revealed distinct functional adaptations. *O. sarsii vadicola* showed enrichment in sensory-related functions, including visual perception, G-protein coupled receptor (GPCR) signaling pathway, and sensory perception of pain (Additional file 2: Table S11). All three ophiuroids shared enrichment in visual and sensory (such as visual perception and GPCR, Additional file 2: Tables S12–S13). *O. sarsii* displayed unique enrichment in sensory perception of temperature stimulus and pain, regulation of growth, response to oxidative stress, stress-activated MAPK cascade, and wound healing (Additional file 2: Table S12). *A. filiformis* showed distinct enrichment in Wnt signaling pathway, wound healing, sensory perception of pain and sound, and response to oxidative stress and bacterium (Additional file 2: Table S13). Chromosomal structure analysis revealed high conservation between *O. sarsii vadicola* and *O. sarsii* compared to *A. filiformis* (Fig. 1C). We identified nine conserved chromosomes (chr) between the two superorders Euryophiurida (*O. sarsii vadicola* and *O. sarsii*) and Ophintegrida (*A. filiformis*) in the order Ophiurida. Several rearrangements of chromosomes were observed, including the fusion of *A. filiformis* chr5, chr11, and part of chr2 into chr1 in the *Ophiura* species, and the split of *A. filiformis* chr1 into chr7 and chr19 in the *Ophiura* species.

### Arm regeneration experiment, morphological observations, and bulk RNA analysis

**Fig. 2:**
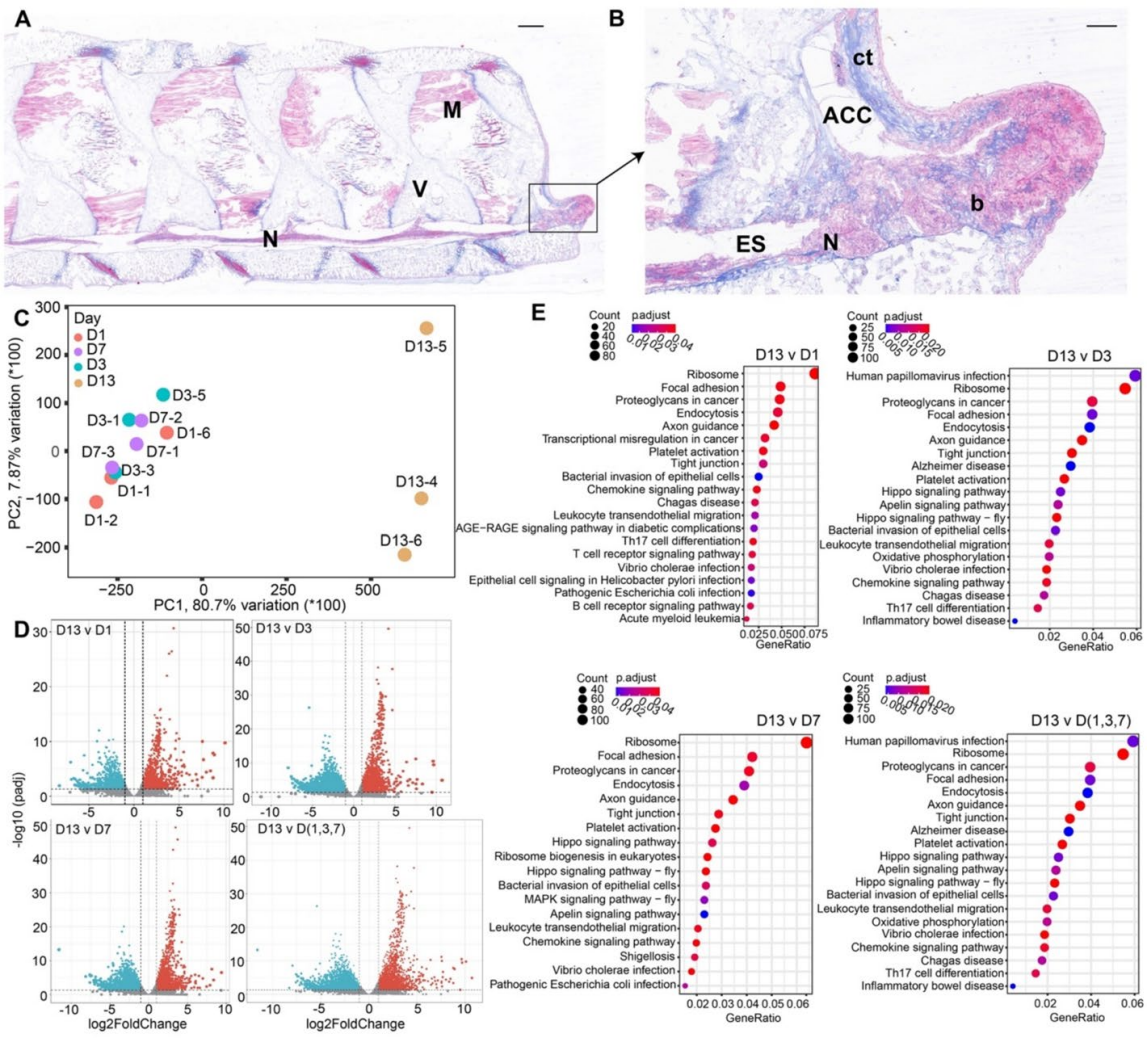
Arm regeneration and differential gene expression in *O. sarsii vadicola* at four post-amputation time points. **A** Histological observations of the arm tip of *O. sarsii vadicola* on day 13 post-amputation. N: nerve tissue, M: muscle tissue, V: vertebral structure. **B** Zoomed-in view of the blastema from **A**. N: nerve tissue, b: blastema, RWC: radial water canal, ACC: aboral coelomic cavity, ct: connective tissue. **C** Principal component analysis (PCA) of gene expression profiles at four time points during regeneration. **D** Up- and downregulated gene expression at day 13 compared to earlier time points. **E** Annotation and enrichment analysis of differentially expressed genes at day 13 post-amputation compared to earlier time points

To identify crucial stages of arm regeneration in *O. sarsii vadicola*, we investigated both morphological changes and gene expression profiles in the distal tissues at four post-amputation time points: spanning wound repair (days 1 and 3) and early regeneration (days 7 and 13). Complete wound healing, observed within 3 days post-amputation (Additional file 1: Fig. S6), marked the transition to early regeneration phase, consistent with previous reports of brittle star regeneration [29]. The wound surface recovered within 1 to 3 days, and an obvious distal blastema was observed by day 13 (Fig. 2A).

Analysis of bulk RNA sequencing data revealed distinct transcriptional patterns. Principal component analysis (PCA) revealed a clear distinction in gene expression profiles between day 13 (D13) samples and earlier time points (Fig. 2C and Additional file 1: Fig. S7). Pairwise comparisons (D13 vs D1, D13 vs D3, D13 vs D7, D13 vs three other groups) were conducted using DESeq2 to identify differentially expressed genes (DEGs) across regeneration stages (Additional file 2: Table S14). Comparisons between day 13 and the earlier three time points (days 1, 3, 7) revealed 2675 significant DEGs, comprising 1149 significantly upregulated and 1526 significantly downregulated genes (Fig. 2D, D13 vs D [1, 3, 7]). The D13 DEGs were predominantly enriched in ribosomal genes, indicating a transition to the tissue growth phase of regeneration. Among these DEGs, we identified 26 genes with known regenerative functions (Additional file 2: Table S14).

Comparative analysis between the stable growth phase (D13) and day 1 revealed 788 significant DEGs, including 423 upregulated and 365 downregulated genes (Fig. 2D). During this critical first day post-amputation, we identified eight regeneration-related DEGs (Fig. 2D, D13 v D1, Additional file 2: Table S14). These included three significantly upregulated genes involved in immunity and wound healing (e.g., *MUC2*) and five upregulated genes enriched in key pathways: endocytosis (ko04144), leukocyte transendothelial migration (ko04670), and T cell receptor signaling pathway (ko04660). This expression pattern indicates robust activation of immune response pathways immediately following amputation. By day 3 (D3), we identified 1823 significant DEGs (Fig. 2D, D13 vs D3), including nine regeneration-related DEGs. Within the 726 significantly upregulated genes, we identified four genes, including *MAP-1B* and *CSRP2*, associated with nervous system development and smooth muscle differentiation. Pathway enrichment analysis revealed disease controls (apelin signaling pathway, ko04371) and cell differentiation/proliferation (Hippo signaling pathway), suggesting concurrent regulation of immune functions and preparation for tissue regeneration on day 3. Day 7 exhibited substantial changes in DEG functionality compared to day 13, with nine regeneration-related genes among 1539 significant DEGs (Fig. 2D). Significantly upregulated genes included *SIK3* and *FAM13A*, which regulate growth and regeneration signals through GPCR and mTOR pathways. Additionally, we observed significant enrichment of the mitogen-activated protein kinases (MAPK) signaling pathway (ko04010), which mediates various cellular functions including proliferation, differentiation, and migration.

### Single-cell transcriptome analysis during regeneration

**Fig. 3:**
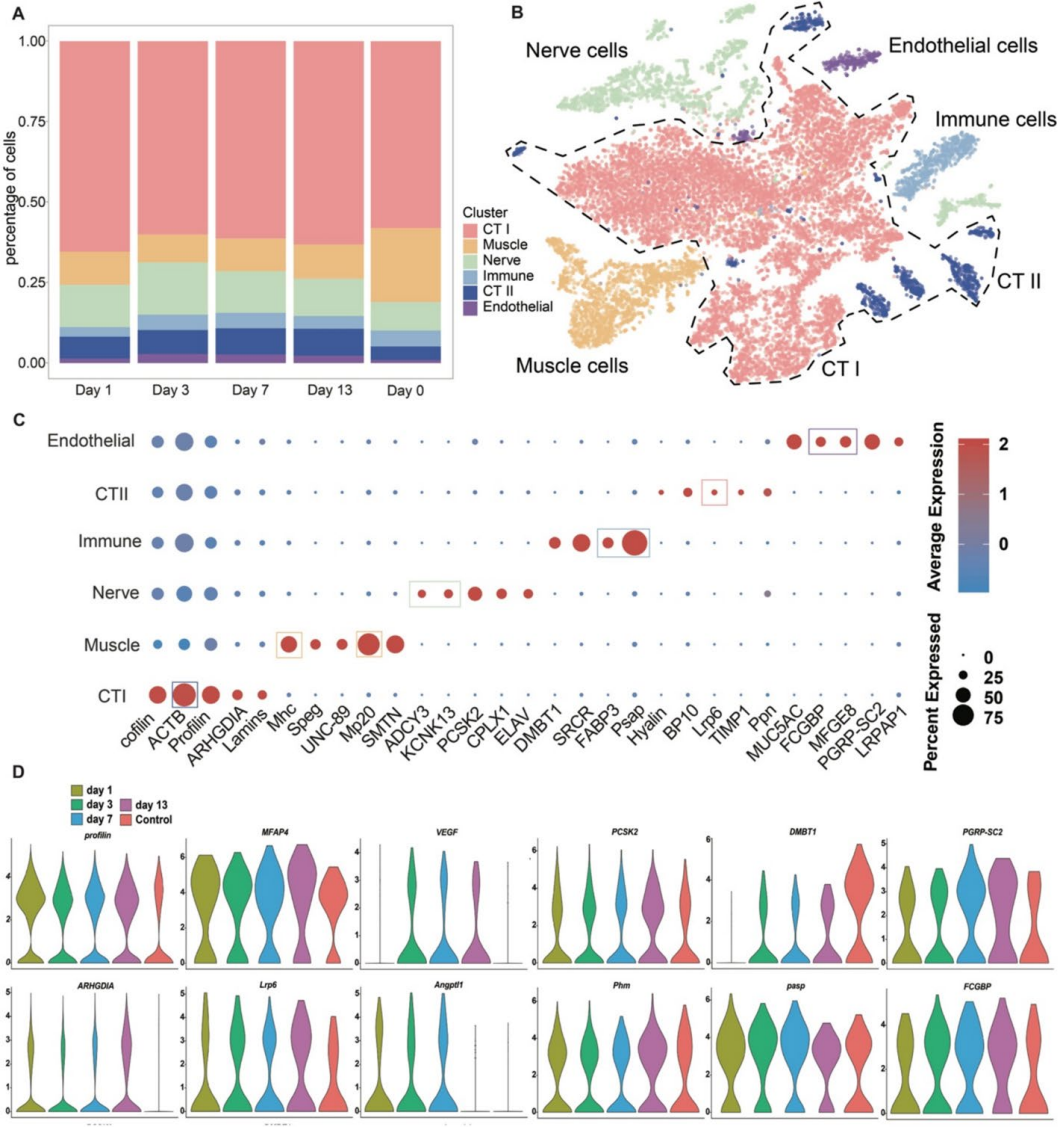
Cell type annotations of *O. sarsii vadicola* arm tips at various time points based on scRNA-seq data. **A** Bar plot showing cell number proportions. **B** *t*-distributed stochastic neighbor embedding (t-SNE) diagram for the single-cell RNA of *O. sarsii vadicola*. Each point represents a cell with circles highlighting CT I and CT II cells. **C** Expression levels of cell type marker genes within the six clusters. Circle sizes indicate the proportion of cells in each cluster expressing a given gene, while the color reflects the mean expression across those cells. **D** Expression levels of marker genes across the six cell clusters at various time points (X-axis), with the Y-axis showing expression levels

To elucidate the cellular composition and dynamics during wound healing and early regeneration, scRNA-seq analysis was conducted for the regenerative arm tissues collected at days 1, 3, 7, and 13 post-amputation, using day 0 wound tissue as the control group. Individual sample cell counts ranged from 3024 (D13) to 5399 cells (D7, Additional file 2: Table S15). After quality filtering and normalization, we retained 21,001 cells for downstream analysis (Additional file 1: Fig. S8 and Additional file 2: Table S16). Using *t*-distributed stochastic neighbor embedding (t-SNE), we identified and annotated six major cell clusters based on marker gene expression profiles (Fig. 3B, Additional file 1: Fig. S9, and Additional file 2: Table S17): two connective tissue clusters (CT I and CT II, marked by *Lrp6* and *ACTB*), muscle cells (marked by *Mhc* and *Mp20* for body movement), nerve cells (marked by *ADCY3* and *KCNK13* for nerve signaling), immune cells (marked by *psap* and *SRCR* for phagocytosis and nonspecific immunity), and endothelial cells (marked by *FCGBP* and *MFGE8* related to water vascular systems).

The CT clusters constituted the largest cell population (68.6% of total cells, Fig. 3A, Additional file 2: Tables S16, S18) and showed significant upregulation of regeneration markers, including *profilin* (Fig. 3C, marker genes of CT I), a known stem cells marker in sea urchins [31]. Detailed analysis revealed two distinct CT subpopulations. CT I was characterized by significant expression of *cofilin* and *ACTB*, along with other actin-regulatory genes [38, 39]. CT II showed active expression during regeneration, with upregulation of developmental/regenerative genes including *BP10* and *Lrp6* [40, 41]. Functional annotation (Additional file 2: Table S17) revealed that CT I was involved in rapid passive post-amputation responses, while CT II activated healing and regeneration processes through the expression of connective tissue-related genes (e.g., *Ppn* and *TIMP1*, Fig. 3C) and regeneration-related genes like *VEGF* and *Hyalin* (Fig. 3C).

We observed distinct temporal patterns in gene expression across regeneration stages. Immune system-related genes, such as *DMBT1*, showed initial suppression followed by increased expression (Fig. 3D). *Profilin* and the actin regulator *ARHGDIA* maintained significant expression during all regeneration phases. *Lrp6* and *VEGF* exhibited gradual increase during the early stage of regeneration (Fig. 3D), suggesting CT II cells’ direct involvement in the regenerative process. Marker genes of neural cell populations, such as *Phm* and *PCSK2*, showed marked upregulation by day 13, suggesting that neural regeneration initiated between days 7 and 13. Additionally, the expression of immune-related genes was affected to varying degrees after amputation. *DMBT1* exhibited transient suppression followed by gradual recovery (Fig. 3D), while the *pasp* gene, which mediates macrophage phagocytic function, showed minimal changes.

**Fig. 4:**
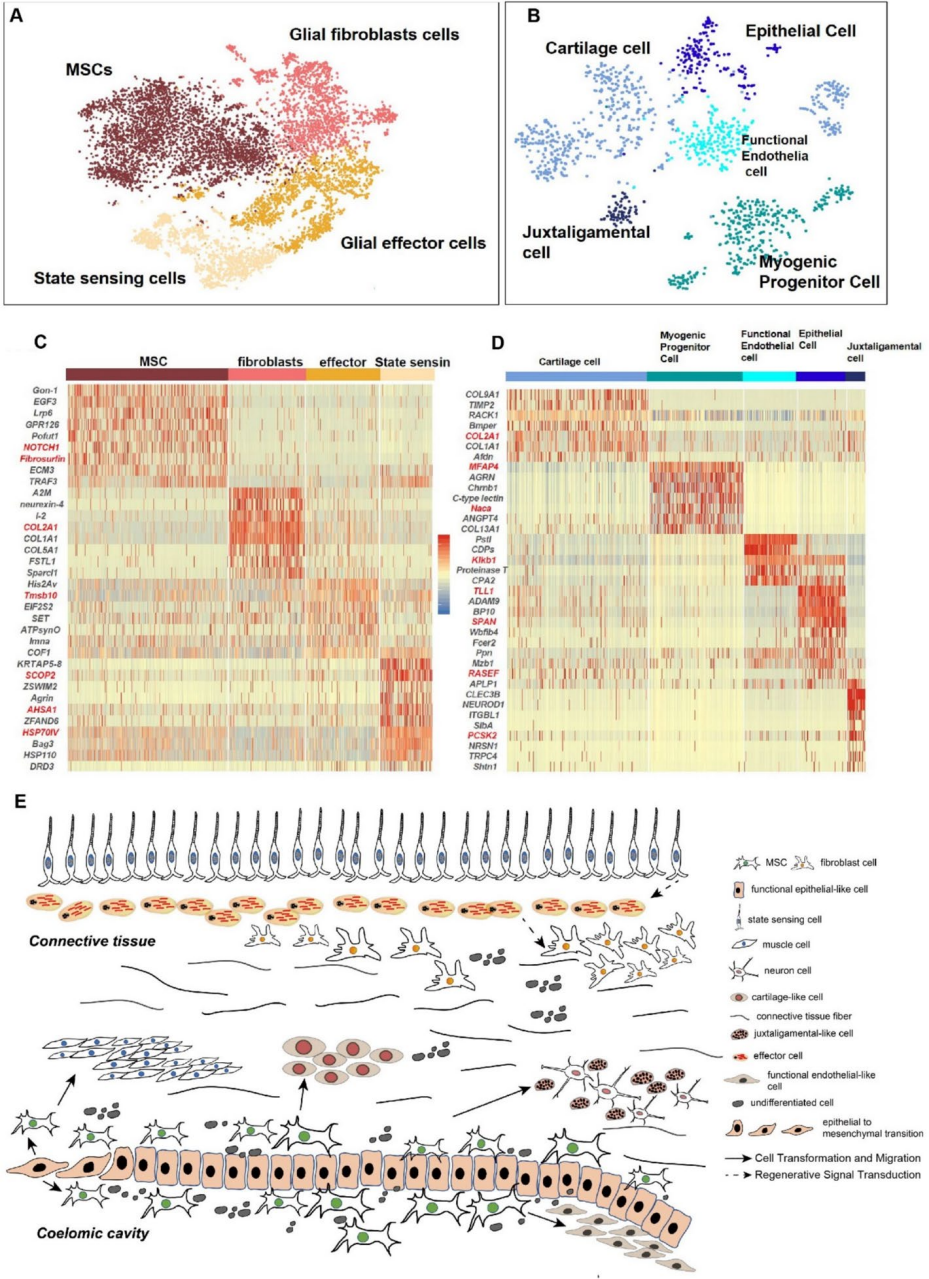
Clustering results and marker gene expression for CT I (**A**, **C**) and CT II (**B**, **D**) cells, with a hypothesis of cellular mechanisms during *O. sarsii vadicola* arm regeneration (**E**)

To further explore the roles of CT I and CT II groups during arm regeneration, we separated these two groups and re-analyzed their expression profiles using t-SNE clustering (Fig. 4A–B). We identified four cell subgroups in CT I cells with relevant marker genes (Additional file 2: Table S19): mesenchymal stem cells (MSCs), glial fibroblasts (marked by various collagens), glial effector cells (marked by actin-related genes), and state sensing cells (Fig. 4C, marked by *HSP70*). In addition, we identified five cell subgroups in CT II cells based on marker genes (Fig. 4D, Additional file 2: Table S20): cartilage cells, myogenic progenitor cells, functional endothelial-like cells, epithelial cells, and juxtaligamental cells. The coordinated activity of diverse CT cells appeared to drive the continuous healing and regeneration in *O. sarsii vadicola* (Fig. 4E). Following receipt of injury signals (NF-κB and TNF-α) by state sensing cells, epithelial cells undergo conversion to mesenchymal stem cells (MSCs). These MSCs regulate cell differentiation and proliferation while migrating along the coelomic and water vascular system endothelium toward the amputation site to establish the blastema. During this migration process, MSCs continuously differentiate into diverse cell types (muscle cells, neuron cells, cartilage-like cells, juxtaligamental-like cells, Fig. 4E, solid arrows) and propagate regenerative signals (Fig. 4E, dashed arrows) through the Wnt/β-catenin signaling pathway by state sensing cells, effector cells, and neuron cells (Fig. 4E).

### Gene-expression dynamics of early-stage regeneration at a single-cell resolution

Several key gene expression events occurred after arm amputation. State sensing cells (marked by *SCOP2* and *HSP70*, Additional file 2: Table S19) initially detected physiological changes, simultaneously activating glial effector cells and glial fibroblasts. This stress response facilitated rapid cellular state transitions. Compared with the control group (day 0), endothelial cell abundance rapidly increased within the first 3 days post-amputation (Fig. 3A). Wound healing was initiated through the expression of fibrinolysis genes, including *Klkb1*, (Additional file 2: Table S20), followed by activation of regenerative signaling pathways, including *NOTCH* and *Wnt/β-catenin*. MSCs, characterized by expression of *NOTCH 1*, *fibrosurfin*, and other genes (Fig. 4C, Additional file 2: Table S19), integrated signals from the *NOTCH*, *Wnt/β-catenin*, and GPCR pathways [19, 41–43], bridging emergency responses and regeneration while regulating cellular differentiation and proliferation [20]. Notably, *fibrosurfin*, crucial for inter-fibrous assembly in connective tissue regeneration [44], showed specific upregulation. Whole-mount in situ hybridization (WMISH) results revealed that *fibrosurfin* expression was primarily in intervertebral regions and the body cavity (Additional file 1: Fig. S10), extending across the regeneration region by day 13.

Regulated by MSCs, four CT II subgroups performed distinct functions (Additional file 2: Table S20). Epithelial cells expressed mucous membrane markers *ADAM9* and *MFGE8* [45, 46], mediating endothelial tissue regeneration through *BP10* expression. Cartilage-like cells, characterized by *COL9A1* and *COL2A1* expression, facilitated ligament and bone regeneration through *COL11A1*. Myogenic progenitor cells, marked by high *AGRN* and *NCNA* expression, may drive muscle and tendon regeneration via *COL13A1*, while functional endothelial-like cells and juxtaligamental cells performed similar neurosecretion functions (*PCSK2*, *Gyc76C*), with endothelial-like cells specifically supporting nerve regeneration through *NRSN* and *Shtn1* expression. To verify key genes expression in CT II subgroups during tissue regeneration, we conducted WMISH analysis targeting the *Lrp6* gene, a myogenic progenitor cell marker. This gene, which encodes a Wnt co-receptor and activates the Wnt signaling pathway through LDL-receptor activity, showed localized expression in muscle tissue at the regenerating arm tip (Additional file 1: Fig. S10).

**Fig. 5:**
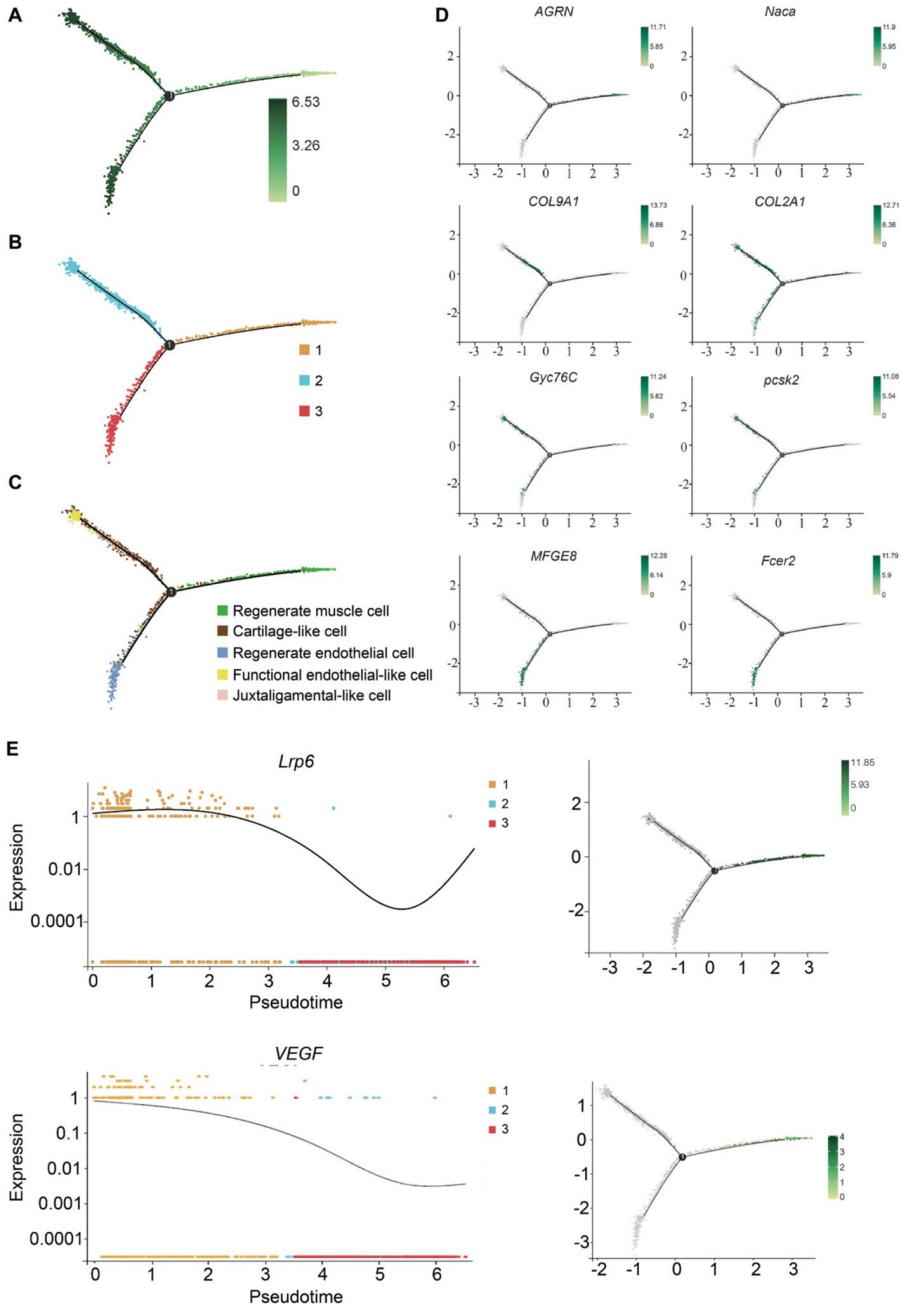
Pseudotiming analysis of CT I and CT II cells during regeneration. **A** Pseudotime ordering of cells. **B** Differentiation status of each branch. **C** Distribution of each cell group along the pseudotime axis. **D** Marker gene expression in cell subgroups over pseudotime. **E** Expression of *Lrp6* and VEGF over pseudotime, with the X-axis showing pseudotime and the Y-axis showing expression levels

To elucidate the sequential progression of tissue regeneration, the Monocle2 program was used to analyze the pseudotemporal ordering of CT II cells (Fig. 5). Integration of pseudotime trajectory (Fig. 5A) and differentiation status plot (Fig. 5B) revealed that the right trajectory branch marks the initiation of differentiation, subsequently diverging into branches 2 and 3 at the differentiation node. Cell population distribution along the pseudotime axis (Fig. 5C) indicates a sequential differentiation pattern: muscle progenitor cells (marked by *AGRN* and *Naca*, Fig. 5D) initiated the process, followed by chondrocyte differentiation, ultimately branching into neural and epithelial tissue regeneration pathways. The first branch involved the expression of cartilage genes (i.e., *COL9A1* and *COL2A1*) and neural markers (i.e., *Gyc76C* and *PCSK2*), while the second branch showed enrichment in mucosal endothelial genes (*MFGE8* and *Fcer2*). Additionally, the mesenchymal stem cell marker *Lrp6* and *VEGF* showed sustained activity during early regeneration (until day 13), coinciding with muscle cell regeneration (Fig. 5E). Validation using Slingshot analysis of CT II cells (Additional file 1: Fig. S11) corroborated the Monocle2 results, confirming muscle progenitor cells as the initiating population, followed by cartilage cell differentiation, before diverging into distinct endothelial cells and juxtaligamental-like cells.

## Discussion

### Chromosome-level genome features in the phylum Echinodermata

The limited genome resources of brittle stars have impeded effective genomic analyses for this group, particularly for cellular and molecular studies related to regeneration. The draft genome of *Ophioderma brevispinum*, reported in 2022, was the first sequenced ophiuroid genome [16]. Recently, two additional ophiuroid genomes were sequenced [34, 35]. Our genome showed higher completeness (94.0%) and comparable assembly quality, with a scaffold N50 of 66.91 Mbp, relative to *O. sarsii* (93.5%, 78.3 Mbp) and *A. filiformis* (92.7%, 68.8 Mbp) (Additional file 2: Table S4). Comparative genomic analysis (Additional file 2: Table S10) revealed that some rapidly evolving gene families, such as GPCR, play essential roles in skeletal [47] and neuronal regeneration [48]. The high-quality *O. sarsii vadicola* genome documented here provides a valuable new resource for studies, particularly in regeneration research.

The three sequenced species represent two superorders: Euryophiurida (*O. sarsii vadicola* and *O. sarsii*) and Ophintegrida (*A. filiformis*). These three genome resources from two superorders provide a valuable foundation for diverse downstream analyses. In this study, analysis of their genomic data revealed similarities and divergences, highlighting adaptations in sensory perception and wound healing post-amputation (Additional file 2: Tables S11–S13). This functional differentiation between *O. sarsii* and *O. sarsii vadicola* revealed key adaptations in temperature response and oxidative stress pathways, likely reflecting response to historical geographical events in their habitats [49]. Population genetics and other molecular techniques will be employed to further reveal the historical dynamics of their divergence and environmental adaptation. We identified several conserved chromosomes and rearrangements across the two superorders in Ophiuroidea. Macrosynteny analysis serves as a powerful tool for tracking orthologous chromosomes across highly divergent species and is increasingly valuable for studying genome rearrangements [50]. Future research should determine the precise timeline of chromosomal rearrangements and investigate the contributions of repeat expansion and chromatin architecture [35] in ophiuroids.

### Organ regeneration at the cellular level in ophiuroids and other echinoderms

Regeneration in ophiuroids has been investigated across species from the orders Amphilepidida, Euryalida, and Ophiurida, with regeneration times ranging from weeks to months [13]. Most studies have centered on *A. filiformis* (superorder Ophintegrida), examining morphological features [14], cellular differentiation [51], and gene expression [52]. A recent genomic and comparative transcriptomic study [35] systematically analyzed gene expression during adult arm regeneration. However, regeneration in other ophiuroid taxonomic groups remains poorly understood, especially given their high morphological and skeletal disparities. Regeneration biology and histology have been documented for the order Euryalida (basket star) in the superorder Euryophiurida [6], highlighting distinct morphogenesis capabilities relative to Ophintegrida. Studies of other groups in Euryophiurida remain limited. Within Ophiurida, although regeneration rates have been reported for some species [13], detailed follow-up studies are lacking. Only one study has examined regeneration in Ophiurida, demonstrating slow regeneration in *Ophionotus victoriae* (family Ophiuridae) through transcriptomic analysis [53] and identifying relevant gene families and pathways, including *Hox*, *Sox*, *Wnt/β-catenin*, *TGF-β*, and *Notch* families.

This study represents the first comprehensive molecular investigation of regeneration in Ophiurida, examining early arm regeneration in *O. sarsii vadicola* through multiple techniques. Distinct changes in both cell types and gene expression profiles were observed during the transition from wound repair (days 1 and 3) to early regeneration (days 7 and 13). The results suggest that regeneration occurs through epimorphosis, involving de-differentiation, differentiation, and proliferation of epithelial and other cell types in the wound and blastema areas (Fig. 5). After distal arm amputation, recovery begins with immune and stress responses from functional endothelial-like cells (*HSP70* and *SCOP2*). Similar to other echinoderms [54, 55], the amputated surfaces of *O. sarsii vadicola* coordinate passive mechanical changes. Effector cells stimulated neural control and muscle activity, increasing collagen fibers produced by fibroblasts to support wound repair, primarily through innate immune responses. While MSCs likely regulate immune function and tissue regeneration [56, 57], no specific *O. sarsii vadicola* MSC genes were identified in this study. During early regeneration, MSCs may regulate immune function through NF-κB and MAPK pathways by inhibiting genes such as *DMBT1* (Additional file 2: Table S19), thereby reducing epithelial differentiation.

The cellular composition during regeneration exhibits considerable complexity. During epimorphosis, both blastema and arm tissues undergo various cellular processes, including differentiation, proliferation, de-differentiation, and re-differentiation [11]. During arm regeneration in ophiuroids, re-epithelialization occurs around the wound site, where endothelial cells transform into MSCs through NF-κB and TNF-α pathways [58, 59]. Morphological studies of ophiuroid and crinoid regeneration suggest that MSCs in the blastema possess pluripotent capabilities and originate from migrating coelomic epithelium [60, 61]. Early calcified spicules initially form within MSCs and are regenerate through specific cell signaling. Concurrently, tissue differentiation is initiated via the Notch and Wnt/β-catenin signaling pathways [62]. Coelomic tissue cells, including muscle cells, also contribute to regeneration by producing cells that differentiate into various tissue types [63, 64]. Muscle cells de-differentiate from myoepithelial cells before re-differentiating [14], whereas in starfish, cavity cells originate from the cavity epithelium [65]. Studies have shown that muscle tissue de-differentiation and regeneration commonly occur in mature echinoderm muscles [10]. De-differentiated muscle cells of the sea cucumber (*Holothuria tubulosa*) contribute to endothelial regeneration in the water vascular system [66], while the presence or absence of longitudinal muscle critically influences planarian regeneration [67]. In our study, muscle regeneration was observed during the initial stage of arm regeneration. However, further investigation is needed to fully understand the potential roles and mechanisms of muscle regeneration in echinoderms.

Connective tissue provides a crucial migratory matrix during blastema and arm tissue regeneration [68]. CT cells emerged as the predominant functional cell type in *O. sarsii vadicola* arm regeneration (Fig. 3). Based on scRNA-seq analyses, we further annotated and identified cell types within this group (Fig. 4) and tracked their dynamics (Fig. 5) to reveal cellular mechanisms. Within CT groups, myogenic progenitor cells served as the primary source of muscle regeneration, responding to Wnt/β-catenin signals from MSCs. Through pseudotime analysis of dynamically expressed marker genes, we found that these myogenic progenitor cells may also initiate tissue differentiation. Moreover, we found that *RACK1* inhibited Wnt/β-catenin signaling (Additional file 2: Table S20), suggesting that the direction of differentiation for each tissue type may depend on the timing of signals to cartilage cells.

### Single-cell sequencing in regeneration studies of model organisms

Our study highlights the essential role of CT cells in ophiuroid arm regeneration, although the original cellular source remains uncertain*.* Connective tissue, comprised extracellular matrices and abundant interconnecting cells, can revert mature cells to a homogeneous progenitor state during regeneration, as demonstrated in forelimb regeneration in the salamander *Ambystoma mexicanum* [33]. Specific connective tissue cell types can regenerate into their corresponding tissues, as observed in periosteal cartilage regeneration at human bone defect sites [69] and mouse muscle tissue regeneration by muscle stem cells and fibroblasts [70]. Similar connective tissue cell types were also identified in *O. sarsii vadicola*, including MSCs, myogenic progenitor cells, and cartilage-like cells. The presence of connective tissue fibers in the regeneration region, as shown in the clustering results (Fig. 3A), further supports the essential role of connective tissues in *O. sarsii vadicola* regeneration.

CT cells appear to serve similar functions in *A. mexicanum* and *O. sarsii vadicola* (Additional file 1: Fig. S12). Our analysis revealed a high similarity between CT cells in this study and those reported in axolotl [71]. Our study produced 21,001 cells for five stages (an average of 4200 cells), close to the output of axolotl limb regeneration (~5000 cells) studies on average [48, 71]. We identified 13 core marker genes, annotated as ribosomal proteins (e.g., *S* and *L* genes, *RPA* and *RPL*), with elevated expression in both species, particularly in *O. sarsii vadicola* (Additional file 2: Table S21). These core markers are conserved across diverse biological processes, including eukaryotic embryo development [72], post-injury neuroregeneration [73], and exhibiting significant heterogeneity across different tissues [74]. Notably, *RPL44* (*evm.TU.CTG.1969.9*) is specifically expressed in mammalian skeletal muscle stem cells [75], highlighting the importance of RP genes in regeneration across both invertebrates and vertebrates. Despite the distant evolutionary relationship between axolotls and ophiuroids, we identified these conserved RP genes as potential key markers for CT cells during regeneration across species, though future experiments are needed to verify their functions.

## Conclusions

This study establishes a chromosome-level genome assembly of *Ophiura sarsii vadicola* and elucidates the cellular and molecular dynamics of arm regeneration in ophiuroids through combined bulk and single-cell transcriptomic profiling. We demonstrate a hierarchical regeneration mechanism characterized by five distinct cell clusters, highlighting the multipotent differentiation of connective tissue-derived progenitor cells into four other lineages. Notably, muscle differentiation emerges as an early-phase event in regeneration, suggesting temporal prioritization of functional tissue reconstruction. These results provide fundamental genomic and cellular insights into echinoderm regeneration and establish an evolutionary framework for understanding marine invertebrate tissue restoration. The high-quality genomic resource and single-cell atlas herein presented offer a benchmark for comparative studies in regenerative biology across metazoans.

## Methods

### Sample collection

*Ophiura sarsii vadicola* specimens used in this study were collected in April 2019 from a cold-water mass (35 m water depth, 35.00 °N and 124.00 °E) in the South Yellow Sea using a benthic trawl. The specimens were maintained in a circulating water system for cultivation. For genome sequencing, arm tissues were immediately preserved in liquid nitrogen and stored at −80 °C. For transcriptome sequencing, specimens were preserved in RNA preservation solution (Tiangen, China).

### Genome sequencing and assembly

High-quality DNA was extracted from an *O. sarsii vadicola* specimen using the cetyl trimethylammonium bromide (CTAB) method. DNA quality and quantity were examined using 1% agarose gel electrophoresis, Qubit 4 instrument (Thermo Fisher Scientific, USA), 2100 Bioanalyzer instrument (Agilent, USA), and qPCR. Multiple libraries were then generated for genome sequencing (Additional file 2: Table S1).

For PacBio sequencing, a SMRTbell library was prepared following the manufacturer’s recommended protocol (Pacific Biosciences, Palo Alto, USA). DNA was randomly fragmented into 20 kbp segments using a Covaris ultrasonicator, followed by enrichment and purification of large DNA fragments with magnetic beads. The fragmented DNA underwent damage and end repair, followed by ligation of stem-loop adapters to both ends. Failed ligations were removed by exonuclease treatment. The library was sequenced on the PacBio Sequel II platform at the Novogene sequencing center (Tianjin, China). For Illumina sequencing, 350 bp DNA libraries were constructed and validated for 150 bp paired-end (PE) sequencing on the HiSeq2000 platform at Novogene. The Illumina reads were trimmed with Trimmomatic v0.33 [76]. Draft genome assembly from short fragments was performed using Jellyfish v2.0 [77] and SOAPdenovo2 [78] with a K-mer size of 17.

PacBio sequencing generated 143.7 Gbp of data with 114.47× coverage and an N50 length of 20,943 bp. Single-pass long reads were preassembled before genome assembly. The longest reads were then selected as seeds and all reads were aligned using DALIGNER [79, 80]. Consensus sequences were generated using the LASort and LAMerge functions of the DALIGNER and pbdacgon programs (https://github.com/pb-cdunn/pbdagcon). The pre-assembled long-read data were assembled into contigs with FALCON v0.3 (https://github.com/PacificBiosciences/falcon3) with the Overlap-Layout-Consensus algorithm. Contigs were polished with Quiver and aligned to 218.32 Gbp of Illumina data (173.92×) using the BWA v0.6 [81]. The final genome assembly was refined using Pilon v.1.24 [82].

For RNA sequencing and genome annotation, total RNA was extracted from skin, arm muscle, podium, and gonad tissues using an animal tissue total RNA extraction kit (Tiangen, China). RNA quality was verified using an Agilent qPCR system. Qualified RNA samples underwent polyA tails enrichment using magnetic beads, followed by fragmentation with NEB Fragmentation Buffer. Fragmented mRNA was converted to Illumina RNA-seq libraries using the NEBNext® Ultra™ RNA Library Prep Kit according to the manufacturer’s instructions. Library quantification was performed using Qubit and fragment length assessment was conducted using a 2100 Bioanalyzer (Agilent, USA). Libraries were sequenced on the Illumina HiSeq2000 platform using the paired-end PE150 strategy at Novogene.

### HI-C sequencing, chromosome-level assembly, and quality control

For HI-C analysis, genomic DNA was extracted from an *O. sarsii vadicola* arm following previously described methods [83]. The HI-C libraries were sequenced on the Illumina HiSeq platform at Novogene, generating 309 Gbp of high-quality data. The HI-C reads were then aligned to the assembled genome using BWA and repetitive and unpaired reads were filtered using Samtools [84]. Reads adjacent to enzyme cut sites were selected to assist genome assembly. The order and orientation of chromosomes were determined (Additional file 2: Table S3) based on contig interaction patterns and locations using Lachesis v180922 [85]. The potential genome assembly contamination was identified using FCS-GX [86] and eliminated.

Genome assembly quality was evaluated through multiple approaches. Illumina reads were aligned to the assembly using BWA. Genome completeness was assessed using two methods: the Core Eukaryotic Genes Mapping Approach (CEGMA) [87], which analyzes 248 conserved genes from six model eukaryotic organisms, and the Benchmarking Universal Single-Copy Orthologs (BUSCO) method [88]. Assembly quality value (QV) was determined using Merqury [89].

### Repeat gene annotation, gene prediction, and functional annotation

Repeats in the assembly were identified using both homology alignment and a de novo approach. Tandem repeats were extracted with the Tandem Repeat Finder (TRF) program via ab initio prediction. Homolog prediction was conducted using RepeatMasker v2.10.0 (https://www.repeatmasker.org/) and associated scripts (e.g., RepeatProteinMask) to extract repeat regions using default parameters. Ab initio prediction generated a de novo repetitive element database using LTR_FINDER v1.0.7 [90], RepeatScout [91], and RepeatModeler (https://www.repeatmasker.org/RepeatModeler/) with default parameters. All repeat sequences with lengths > 100 bp and gaps of “N”s making up less than 5% were retained in the raw transposable element (TE) library. DNA-level repeat identification used RepeatMasker with both Repbase (https://www.girinst.org/repbase) and our de novo TE library.

Protein-coding genes were predicted (Additional file 2: Table S6) using an integrated pipeline incorporating Augustus v3.2.3 [92], Geneid v1.4 (https://genome.crg.cat/software/geneid/), Genescan v1.0 (https://hollywood.mit.edu/GENSCAN.html), GlimmerHMM v3.04 (https://ccb.jhu.edu/software/glimmerhmm/), and SNAP v2013-11–29 [93]. Homologous proteins from Ensembl (https://uswest.ensembl.org/index.html) and NCBI (https://www.ncbi.nlm.nih.gov/protein) databases were aligned to the genome using tblastn v2.2.26 (*E* value ≤ 1e^−5^), followed by generation of spliced alignments using GeneWise v2.4.1 (https://www.ebi.ac.uk/Tools/psa/genewise/). RNA-seq reads were aligned using Hisat2 v2.0.4 [94] and assembled into genome-based transcripts using Cufflinks v2 [95], both with default parameters.

Gene function annotation involved multiple approaches. We performed BLASTp search against the Swiss-Prot database (https://www.uniprot.org/) with an *E* value of ≤1e^−5^. InterProScan v5.31 program (https://github.com/ebi-pf-team/interproscan) was used to annotate motifs and domains through comparison with multiple databases: ProDom (https://prodom.prabi.fr/prodom/current/html/home.php), PRINTS (https://130.88.97.239/PRINTS/index.php), Pfam (https://pfam.xfam.org/), SMART (https://smart.embl-heidelberg.de/), PANTHER (https://www.pantherdb.org/), and PROSITE (https://prosite.expasy.org/). Gene Ontology (GO) IDs were assigned from InterPro entries (https://www.ebi.ac.uk/interpro/). Additional protein functions were annotated based on the top BLAST hits in the nr database (*E* values of <10^−5^) and the Kyoto Encyclopedia of Genes and Genomes (KEGG) database. tRNAs were identified with tRNAscan-SE v2.0 [96]. rRNA identification used BLAST searches against sequences from related species, given their high conservation. Other non-functional RNAs, including miRNAs and snRNAs, were identified using the Rfam database and INFERNAL v1.1.5 [97].

### Regeneration culture experiments and tissue sampling over time

Complete and healthy *O. sarsii vadicola* individuals were acclimated in laboratory conditions for 1 month before experimentation. Ten individuals were housed in each of five 20-L tanks maintained at 12 °C and salinity of 31 psu. Following acclimation, two arm tips per individual were amputated at 13, 7, 3, and 1 days before tissue collection. After amputation, ophiuroids were returned to their respective tanks. Regeneration tissue samples (~1 cm) were collected from the wound edge toward the central disk using sterilized scissors and processed for multiple analyses: 1) for scRNA-seq, tissues from three individuals per time point were enzymatically digested; 2) for bulk RNA sequencing, regenerative distal tissues for three individuals per tank were flash-frozen in liquid nitrogen and preserved at −80 °C; 3) for histology and in situ hybridization, the remaining samples were fixed overnight at 4 °C in 4% PFA prepared in 1× PBT reagent (phosphate-buffered saline with 0.1% Tween-20), washed in 1× PBT, and stored in 100% methanol at −20 °C.

### Single-cell RNA sequencing and bulk RNA sequencing

To obtain sufficient live cells for scRNA-seq analysis, regenerative tissues from four individuals were pooled per time point, sampling at 13, 7, 3, 1, and 0 days post-amputation.

Single-cell capture was performed using a Chromium Controller Instrument (10× Genomics; Pleasanton, CA). A 1-cm section of arm tissue was excised and immediately digested in 0.1 mg/mL collagenase 1 (Invitrogen, USA) for 30 min at 37 °C. Digestion was stopped using DMEM/F12 with 15% FBS. Dissociated cells were collected by centrifugation (1300 rpm, 4 °C, 5 min). The cell suspension was filtered through a 40-μm filter (Greiner, Germany) and resuspended in PBS with 1% bovine serum albumin (BSA). Cells were then collected by centrifugation at 4 °C. After centrifugation, cell count and concentration were determined using a Countess II Automated Cell Counter and adjusted to 1000 cells/μL before library preparation.

According to the manufacturer’s instructions, cell suspensions were processed using a Chromium Controller Instrument (10× Genomics, Pleasanton, CA, USA). Single-cell gel bead emulsions (GEMs) were generated using the Single Cell 3’ library and Gel Bead Kit v2 (10× Genomics, 120237). Full-length barcode cDNAs were then PCR-amplified. The final Single Cell 3’ library comprised P5 and P7 primers for PCR amplification. Barcoded sequencing libraries were quantified using standard curve qPCR and Agilent Bioanalyzer 2100. Finally, sequencing was performed on a NovaSeq 6000 platform. The library preparation and sequencing were conducted at the Gene Denovo sequencing center (Guangzhou, China). Bulk RNA sequencing of regenerated tissues followed the same RNA extraction, library preparation, and sequencing protocols described in the genome assembly section above and was conducted at Novogene.

### Divergence time analysis

We analyzed genomic data from nine echinoderm species (Additional file 2: Table S9), including two ophiuroids [34, 35], to investigate the phylogenetic relationship of *O. sarsii vadicola*. After filtering genomic repeats using TBtools [98], we extracted representative CDS and peptide sequences from the filtered annotations. Using the OrthoFinder v2.3.14 [99], we clustered the peptide sequences into ortholog groups. A total of 1537 single-copy genes were selected for phylogenetic relationship reconstruction. We identified the optimal substitution model, LG+F+R7, using ModelFinder [100]. We then performed maximum likelihood (ML) analysis using IQ-TREE v1.6.12 [101] with 1000 bootstrap replicates. For molecular dating, we used a fossil calibration point between *Apostichopus japonicus* and *Holothuria leucospilota* (CI: 308.8–157.8 Mya, median: 233 Mya, adjusted time: 250 Mya) obtained from TIMETREE5 [102–104]. Using the ML tree topology and fossil calibrations, we estimated divergence times with the MCMCtree program embedded in PAML [105]. The analysis employed an independent rates clock model with the root set at the deuterostome-protostome split (RootAge < 700 Mya). We used LG.dat as the amino acid substitution rate file and specified the following MCMC parameters: burnin = 2500, sampfreq = 100, and nsample = 10,000.

### Comparative genomic analyses

From the OrthoFinder results, we selected 27,305 gene families that contained genes present in at least 50% of the analyzed species. Using CAFÉ v4 [106], we analyzed these orthogroups to estimate gene family expansions and contractions based on our divergence time in the phylogenetic tree. We then annotated rapidly evolved gene families (*p* < 0.05) using eggNOG-mapper2 and assessed enrichment using ClusterProfiler v4 [107]. To identify core and ophiuroid-specific gene families, we analyzed orthogroups from *O. sarsii vadicola*, *O. sarsii*, and *A. filiformis* using OrthoVenn2 [108]. These orthogroups were functionally annotated using eggNOG-mapper2 [109] and GO enrichment analysis was performed using clusterProfiler v4 [107]. For synteny analysis between *O. sarsii vadicola* and the two closely related ophiuroids, we performed reciprocal BLAST searches (*E* value < 1e^−10^) using complete protein sequences to identify putative orthologs and visualize collinearity among the three species.

### Bulk RNA sequence analysis

Raw reads for the bulk RNA sequence analysis were pre-processed and filtered using Trimmomatic v0.36 [76], yielding the clean read dataset. Clean reads were then aligned to the *O. sarsii vadicola* reference genome using Hisat2 v2.0.5 [94]. The alignment results were then compressed and sorted using Samtools (Additional file 2: Table S14), and expression quantification was performed using the FeatureCounts program to obtain read count data. The fragments per kilobase of exon model per million mapped fragments (FPKM) value was then calculated for each gene.

Differential expression analysis was conducted using DESeq2 v1.34.0 [110] based on the read count matrix to determine differentially expressed genes between time points. Thresholds including a *p* value ≤ 0.01 and |log_2_ (fold change)| ≥ 1 were used to identify genes that were significantly differentially expressed. Inter-sample correlation analysis (correlation) was performed using the “Cor” function in R (v4.0.0) and correlations were plotted using the pheatmap package (https://CRAN.R-project.org/package=pheatmap), hcluster for sample clustering, and PCAtools (https://github.com/kevinblighe/PCAtools) for principal component analysis. The expression of key functional genes in the early stages of wound healing and regeneration was analyzed alongside annotations of significantly differentially expressed genes to assess their roles and expression trends in the process. These results were visualized using the EnhancedVolcano v1.12.0 package (https://github.com/kevinblighe/EnhancedVolcano). KEGG enrichment analysis of gene function was performed using significantly differentially expressed genes (*p* value ≤ 0.01 and |log_2_FC| ≥ 1) across different time points with ClusterProfiler v4.0.2.

### scRNA-seq data analysis

We used Cell Ranger v3.1.0 (https://support.10xgenomics.com/single-cell-gene-expression/software/pipelines/latest/installation) for initial data processing, including FASTQ file generation (mkfastq function), read quantification (count function), and data aggregation (aggr function) to generate gene expression matrices for individual cells. Using the Seurat v4.0.1 [111], we performed cell filtering based on the *O. sarsii vadicola* genome, followed by subsequent data standardization.

For the five samples, we filtered cells using the following criteria: gene count per cell (>3000), UMI count per cell (>10,000), and mitochondrial gene percentage (>20%). We standardized the data using SCTransform and performed dimensionality reduction through principal component analysis (PCA). Cell clustering was performed using the first 30 PCA components with the shared-nearest neighbor (SNN) method at an optimal resolution parameter of 0.4. We visualized dimensionality reduction results using Clustree [112] for initial exploratory analysis. To determine the optimal SNN resolution (0.4), we conducted merged contour analysis for each time point, balancing expected cluster numbers based on known marker expression with the largest average contour width. We initiated clustering at a resolution of 0.03 and employed t-SNE for visualization. For differential expression analysis, we used the “FindAllMarkers” function with a likelihood ratio test. We identified differentially expressed genes using three criteria (1) *p* value ≤ 0.01; (2) log_2_ (fold change [FC]) ≥ 0.25; and (3) gene detection in >25% of cells within a specific cluster. We then performed GO [113, 114] and KEGG pathway [115] enrichment analyses to characterize the primary functions of each cluster. To further elucidate the functions of significantly differentially expressed genes and assist in cell type identification, we consulted multiple databases: Universal Protein (UniProt, https://www.uniprot.org/), Single Cell Expression Atlas (https://www.ebi.ac.uk/), The Human Protein Atlas (https://www.proteinatlas.org/), and Echinobase Home (https://www.echinobase.org/echinobase/).

We analyzed single-cell trajectories and gene expression patterns of CT II using the Monocle v2.8.0 [116]. This analysis reduces dimensional space to one or two dimensions for cell sorting, revealing tree-like structures with distinct branches and tips. We identified divergently expressed genes using a false discovery rate (FDR) threshold of <1e^−5^. For pseudotime analysis, we used Slingshot v2.2.0 [117] with default parameters. Slingshot constructed a minimum spanning tree (MST) based on cellular subpopulations to estimate global lineage structure. Differentiation trajectories were fitted using simultaneous principal curves and orthogonal projection methods, generating smooth differentiation trajectories with corresponding pseudotime values.

For cross-species comparisons between axolotls and ophiuroids regeneration capabilities, we analyzed the axolotl limb bud tissue dataset [71]. We first filtered the data using Seurat v4.3.0, retaining cells with UMI counts between 1000 and 25,000, resulting in 4113 normalized cells. We identified highly variable genes for PCA using 50 principal components (number of principal components, npcs). Using the PCA results, we performed cell neighbor identification and clustering at a resolution of 0.6. Cell type annotation of CT was based on marker gene expression in these clusters, followed by repeated dimensionality reduction and clustering. The core marker genes were identified using two criteria: adjusted *p* values < 0.05 and log fold change > 0.25. For dataset integration between the axolotl and *O. sarsii vadicola*, we first conducted homologous protein sequences alignment using BLASTp v.2.2.28+. We retained 11,608 one-to-one homologous gene pairs present in the single-cell data, identifying 8984 gene pairs. Using these pairs, we conducted canonical correlation analysis (CCA) to integrate axolotl CT cells with ophiuroid CT I and CT II cells to remove batch effects. Finally, we visualized gene expression patterns between species using Uniform Manifold Approximation and Projection (UMAP).

### Connective tissue and immunohistology observations

We analyzed paraffin-embedded connective tissue samples from each regeneration stage using Masson’s trichrome staining and immunohistology (IHC). We sectioned the embedded tissues to 10-μm thickness. For Masson’s trichrome staining, we used a commercial kit (Solarbio, Beijing, China) following the manufacturer’s protocols to differentiate connective tissue fibers (blue) from muscle fibers (red). IHC was performed with a standard protocol (ZSGB-BIO, OriGene Technologies, Inc., China). After deparaffinization and antigen retrieval, we blocked endogenous peroxidase activity using 3% hydrogen peroxide. For antibody blocking, we used normal rabbit serum for goat-derived antibodies and BSA for antibodies from other sources. For antibody treatment, we incubated sections with the primary antibody overnight at 4 °C. We then washed the sections in PBS (pH = 7.4) with agitation. We incubated the sections with HRP-labeled secondary antibody specific to *Ophiura sarsii vadicola* at room temperature for 50 min.

For visualization, we applied DAB color-developing solution and monitored color development microscopically, with brownish-yellow indicating positive signals.

We stopped the reaction with tap water and proceeded with counterstaining. The sections were counterstained with hematoxylin for 3 min, followed by treatment with hematoxylin differentiation solution and then the bluing solution, with running water washes between steps.

The final dehydration process involved sequential treatment with increasing concentrations of ethanol (75%, 85%, 100%), followed by anhydrous ethanol, n-butanol, and xylene for 5 min each. After brief air-drying, we mounted the sections with neutral gum. We visualized and photographed the stained sections with a Nikon Eclipse Ci microscope. Successfully stained sections showed blue nuclei from hematoxylin staining and brownish-yellow signals indicating a positive DAB reaction.

### In situ hybridization

We performed in situ hybridization on arm regeneration sections using DIG-labeled antisense probes for *Lrp6* and *fibrosurfin* (Additional file 2: Table S22), following a published protocol [118]. We first rehydrated sections through graded ethanol (70%, 50%, and 30%) and washed them three times in 1× MABT buffer (0.1 M maleic acid [pH = 7.5], 0.5 M NaCl, 0.1% Tween-20). After proteinase K digestion for 5 min, we stopped the reaction with 4% paraformaldehyde in 1× PBS for 20 min, followed by 5-min washes in 1× PBT (1× PBS and 0.1% Tween-20). The sections were pre-treated with HB buffer (50% deionized formamide, 10% PEG, 0.05 M NaCl, 0.1% Tween-20, 0.005 M EDTA, 0.02 M yeast Tris pH 7.5, 0.1 mg/ml yeast tRNA, 1× Denhardt’s solution, and DEPC-treated water) at 50 °C for 1 h. Sections were then incubated with HB containing 0.2 ng/μL antisense probe for 8 days at 50 °C, followed by further incubation in fresh HB without probe for 3 h at 50 °C, and then washed once in MABT at 50 °C and once at room temperature. We then performed three washes in 0.1× MABT and one in 1× MABT before overnight incubation in anti-DIG AP antibody (1:1000) at 4 °C. After five MABT washes and two alkaline phosphatase buffer washes, we performed chromogenic detection for 3–4 h. The reaction was stopped with 1× MABT containing 0.05 M EDTA, followed by three MABT washes. Specimens were stored in 50% glycerol at 4 °C and imaged using an Olympus SZX16 microscope (Olympus Corporation, Japan).

## Supplementary Information

Additional file 1: Figures S1–S12 

Additional file 2: Tables S1–S22 [122–133]

## Data Availability

The data of genome sequencing and the genome assembly data of Ophiura sarsii vadicola in this study have been deposited at NCBI under the BioProject PRJNA732836 [119]. The bulk RNA sequencing data and single-cell RNA sequencing data have been deposited at NCBI under BioProject PRJNA942107 [120]. Other echinoderm genomes applied in comparative analyses are listed in Additional File 2: Table S4. The cell expression information, bulk RNA expression data, histology photos relevant to arm regeneration and the script for across-species analysis used in this study, along with two additional files, are available in FigShare [121].
