# Supplementary material for: Chromosome-level genome assembly and single-cell analysis unveil molecular mechanisms of arm regeneration in the ophiuroid Ophiura sarsii vadicola
Source: Genome Biol. 2025 Mar 31;26:82. doi: 10.1186/s13059-025-03542-5 (PMC11959729; doi:10.1186/s13059-025-03542-5)
Supplement: Supplementary file 2 — Additional file 2: Tables S1–S22 [122–133] [file 13059_2025_3542_MOESM2_ESM.docx]

**Chromosome-level genome assembly and single-cell analysis unveil molecular mechanisms of arm regeneration in the ophiuroid *Ophiura sarsii vadicola***

**Supplementary tables**

Table S1 Statistics of genome sequencing data of *Ophiura sarsii vadicola*.

Table S2 The results of genome assembly.

Table S3 The results of sequences matching to the chromosome.

Table S4 The information for known genome assembly of class Echinodermata.

Table S5 Repetitive sequences in the *Ophiura sarsii* vadicola genome.

Table S6 The coverage and mapping rate of short reads aligned to the genome.

Table S7 The quality assessment results of the assembled genome.

Table S8 Summary of protein-coding gene prediction in *Ophiura sarsii vadicola* genome.

Table S9 The comparison of gene structure and gene numbers for echinoderms used for comparative analyses.

Table S10 GO enrichment analysis of significant expanded gene families.

Table S11 GO enrichment results of *Ophiura sarsii vadicola* specific orthogroups.

Table S12 GO enrichment results of *Ophiura sarsii* specific orthogroups.

Table S13 GO enrichment results of *Amphiura filiformis* specific orthogroups.

Table S14 Significantly differentially expressed key genes during regeneration using RNA sequencing data.

Table S15 Statistics of sing-cell RNA sequencing data comparison results.

Table S16 Cell number and intracellular gene content of single-cell RNA sequencing data (21,001 cells).

Table S17 Marker gene information of different tissue and cell groups.

Table S18 Statistical table of cell numbers for each cell cluster at different regeneration stage.

Table S19 Marker gene information of each cell group in CT Ⅰ.

Table S20 Marker gene information of each cell group in CT Ⅱ.

Table S21 Expression of core markers (between axolotl and ophiuroid) in *Ophiura sarsii vadicola*.

Table S22 The sequence information for probes used in the whole mount in situ hybridization experiment.

**Table S1 Statistics of genome sequencing data of *Ophiura sarsii vadicola*.**

| Pair-end libraries | Insert size (bp) | Total data (G) | Read length (bp) | Sequence coverage (×) |
| --- | --- | --- | --- | --- |
| Illumina short reads | 350 | 218.32 | 150 | 173.92 |
| Pacbio long reads | - | 143.70 | - | 114.47 |
| Hi-C library | 350 | 309 | 150 | - |

Note: “-” means no data.

**Table S2 The results of genome assembly.**

|  | Contig length (bp) | Scaffold length (bp) | Contig number | Scaffold number |
| --- | --- | --- | --- | --- |
| Total | 1,267,941,585 | 1,268,118,785 | 1,988 | 216 |
| Max | 13,471,353 | 147,934,811 | - | - |
| Number >= 2000 | - | - | 1,988 | 216 |
| N50 | 2,259,232 | 66,915,169 | 163 | 8 |
| N60 | 1,884,935 | 62,702,253 | 225 | 10 |
| N70 | 1,467,893 | 56,980,601 | 301 | 12 |
| N80 | 977,516 | 52,689,219 | 404 | 15 |
| N90 | 496,165 | 51,704,667 | 584 | 17 |

Note: “-” means no data.

**Table S3 The results of sequences matching to the chromosome.**

| Sequence ID | Cluster number | Sequence length (bp) |
| --- | --- | --- |
| Hic_asm_0 | 139 | 147,934,811 |
| Hic_asm_1 | 85 | 92,748,282 |
| Hic_asm_2 | 113 | 80,758,857 |
| Hic_asm_3 | 88 | 70,437,490 |
| Hic_asm_4 | 73 | 68,898,335 |
| Hic_asm_5 | 65 | 67,708,752 |
| Hic_asm_6 | 82 | 66,966,678 |
| Hic_asm_7 | 76 | 64,701,964 |
| Hic_asm_8 | 71 | 52,984,193 |
| Hic_asm_9 | 64 | 56,980,601 |
| Hic_asm_10 | 126 | 54,509,896 |
| Hic_asm_11 | 96 | 52,689,219 |
| Hic_asm_12 | 130 | 52,573,618 |
| Hic_asm_13 | 102 | 49,102,080 |
| Hic_asm_14 | 69 | 58,363,436 |
| Hic_asm_15 | 110 | 62,702,253 |
| Hic_asm_16 | 88 | 66,915,169 |
| Hic_asm_17 | 100 | 51,704,667 |
| Hic_asm_18 | 112 | 44,580,623 |
| Total scaffold placed | 19 | 1,263,260,924 (99.62%) |
| Total scaffold unplaced | 197 | 4,857,861 (0.38%) |

**Table S4 The information for known genome assembly of class Echinodermata.** Number after the Bioproject number, citation number (details in main text).

| Genome assembly statistics | *Ophiura sarsii vadicola* | *Ophiura sarii* | *Amphiura filiformis* | *Ophionereis fasciata* | *Ophiothrix spiculata* | *Acanthaster planci* | *Pisaster ochraceus* | *Asterias rubens* | *Strogylocentrotus purpuratus* | *Lytechinus variegatus* | *Holothuria leucospilota* | *Apostichopus japonicus* | *Anneissia japonica* |
| --- | --- | --- | --- | --- | --- | --- | --- | --- | --- | --- | --- | --- | --- |
| Size (Mb) | 1,268.11 | 1,592.18 | 1,570.00 | 1,184.53 | 2764.32 | 383.69 | 401.94 | 417.60 | 921.86 | 870.40 | 1,390.00 | 804.62 | 589.63 |
| GC% | 37.21 | 37.11 | 36.67 | 32.60 | 35.10 | 41.50 | 40.35 | 38.77 | 37.40 | 36.31 | NA | 36.80 | 35.00 |
| Predicted heterozygosity | 1.80% | 1.97% | 3.22% | NA | NA | NA | NA | NA | NA | 2.85% | 2.11% | 1.59% | NA |
| Number of scaffolds | 216 | 986 | 597 | 3,968,282 | 75,696 | 1,766 | 1,844 | 150 | 871 | 104 | 2,312 | 3,278 | 76,727 |
| Number of contigs | 1,988 | 8,048 | 2,057 | 5,719,793 | 644,798 | 18,089 | 106,530 | 621 | 1,546 | 466 | 2,318 | 4,741 | 118,759 |
| N50 length (scaffolds) | 66.91 Mb | 78.03 Mb | 68.86 Mb | 484.00 bp | 72.78 Kb | 1.52 Mb | 20.19 Mb | 20.56 Mb | 2.05 Mb | 45.60 Mb | 56.10 Mb | 487.24 Kb | 19.05 Kb |
| Chromosome number | 19 | 19 | 20 | NA | NA | NA | 22 | 22 | NA | 19 | 23 | NA | NA |
| Number of Proteins | 26,010 | 27,099 | 30,267 | NA | NA | 24,323 | NA | 24,049 | 23,300 | 27,232 | 36,089 | 30,221 | 26,762 |
| BioProjects | PRJNA732836 (This study) | PRJNA1057532(123) | PRJNA1029566(124) | PRJEB10339(125) | PRJNA182997(126) | PRJDB3175(127) | PRJNA532896 (128) | PRJEB33974 (129) | PRJNA10736 (130) | CNP0000959 (131) | PRJNA747844 (132) | PRJNA354676 (133) | PRJNA615663 (134) |

**Table S5 Repetitive sequences in the *Ophiura sarsii vadicola* genome.**

|  | **Denovo+Repbase** | | **TE Proteins** | | **Combined TEs** | |
| --- | --- | --- | --- | --- | --- | --- |
|  | Length (bp) | % in Genome | Length (bp) | % in Genome | Length (bp) | % in Genome |
| DNA | 10,792,821 | 0.85 | 1,260,807 | 0.10 | 11,931,087 | 0.94 |
| LINE | 9,298,259 | 0.73 | 36,284,619 | 2.86 | 40,424,318 | 3.19 |
| SINE | 1,527,952 | 0.12 | 0 | 0.00 | 1,527,952 | 0.12 |
| LTR | 617,361,905 | 48.69 | 8,617,978 | 0.68 | 618,074,641 | 48.75 |
| Unknown | 64,595,198 | 5.09 | 0 | 0.00 | 64,595,198 | 5.09 |
| Total | 694,776,754 | 54.80 | 46,160,474 | 3.64 | 702,260,284 | 55.39 |

**Table S6 The coverage and mapping rate of short reads aligned to the genome.**

| Coverage and mapping rate |  | Percentage |
| --- | --- | --- |
| Reads | Mapping rate (%) | 96.62 |
| Genome | Average sequencing depth | 143.30 |
|  | Coverage (%) | 99.59 |
|  | Coverage at least 20× | 98.83 |

**Table S7 The quality assessment results of the assembled genome.**

|  | The number of Assembled core gene | Completeness percentage (%) |
| --- | --- | --- |
| CEGMA | 233 | 93.95 |
| BUSCO notation assessment results (genome) | C:94.2% [S:93.2%, D:1.0%], F:0.9%, M:4.9%, n:954 | |
| BUSCO notation assessment results (protein) | C:94.0% [S:92.1%, D:1.9%], F:1.8%, M:4.2%, n:954 | |

Note: C, the present of complete genome compared with BUSCO data set; S, the present of single copy genes; D, the present of duplicated genes; F, the present of Fragmented genes; M, the present of Missing genes, and n is the number of genes in BUSCO data set

**Table S8 Summary of protein-coding gene prediction in *Ophiura sarsii vadicola* genome.**

|  | Gene set | Number | Average transcript length (bp) | Average CDS length (bp) | Average exons per gene | Average exon length (bp) | Average intron length (bp) |
| --- | --- | --- | --- | --- | --- | --- | --- |
|  | Augustus | 32,979 | 15,517.89 | 1,413.45 | 6.07 | 232.93 | 2,782.97 |
|  | GlimmerHMM | 87,131 | 13,151.40 | 656.93 | 4.02 | 163.52 | 4,140.73 |
|  | SNAP | 48,908 | 37,745.78 | 1,106.35 | 6.25 | 176.91 | 6,974.10 |
|  | Geneid | 33,290 | 20,968.78 | 1,426.19 | 5.68 | 250.96 | 4,173.15 |
| De novo | Genscan | 32,231 | 25,256.48 | 1,582.66 | 6.87 | 230.22 | 4,029.89 |
|  | Aja | 18,283 | 7,379.57 | 823.26 | 3.65 | 225.40 | 2,471.73 |
|  | Spu | 15,493 | 12,125.12 | 1,120.43 | 5.19 | 216.03 | 2,628.67 |
| Homolog | Apl | 14,728 | 15,082.87 | 1,267.80 | 5.96 | 212.78 | 2,786.21 |
|  | PASA | 36,789 | 19,021.59 | 1,157.00 | 5.37 | 215.39 | 4,086.46 |
| RNASeq | Transcripts | 132,519 | 13,541.66 | 1,989.18 | 3.86 | 515.68 | 4,043.02 |
| EVM | | 31,834 | 17,804.45 | 1,394.50 | 6.22 | 224.08 | 3,141.66 |
| Pasa-update* | | 31,452 | 18,912.78 | 1,422.66 | 6.34 | 224.52 | 3,277.44 |
| Final set* | | 26,226 | 22,018.04 | 1,583.07 | 7.23 | 218.82 | 3,277.68 |

#Aja: *Apostichopus japonicus*; Spu: *Strogylocentrotus purpuratus*; Apl: *Acanthaster planci*

**Table S9 The comparison of gene structure and gene numbers for echinoderms used for comparative analyses.**

| Species | Number | Average transcript length (bp) | Average CDS length (bp) | Average exon length (bp) | Average intron length (bp) |
| --- | --- | --- | --- | --- | --- |
| *Ophiura sarsii vadicola* | 26,226 | 22,018.04 | 1,583.07 | 218.82 | 3,277.68 |
| *Ophiura sarsii* | 27,099 | 25,854.00 | 1,586.29 | 318.67 | 2,893.00 |
| *Amphiura filiformis* | 30,267 | NA | 1518.70 | NA | NA |
| *Asterias rubens* | 24,049 | 2,928.00 | 2,146.00 | 297.00 | 1,650.00 |
| *Acanthaster planci* | 24,323 | 17,773.46 | 2,105.21 | 188.23 | 1,538.46 |
| *Lytechinus variegatus* | 27,232 | 3,280.00 | 2,197.00 | 335.00 | 2,297.00 |
| *Strongylocentrotus purpuratus* | 23,300 | 17,783.86 | 2,076.47 | 198.15 | 1,657.05 |
| *Holothuria leucospilota* | 36,089 | 19,136.00 | 1,542.00 | 229.00 | 3,117.00 |
| *Apostichopus japonicus* | 30,350 | 7,791.25 | 1,181.37 | 193.57 | 1,295.26 |
| *Anneissia japonica* | 25,410 | 2,791.00 | 1,804.00 | 327.00 | 1,702.00 |

**Table S10 GO enrichment analysis of significant expanded gene families.**

| Gene family ID | Annotation |
| --- | --- |
| OG0000003 | scavenger receptor activity |
| OG0000024 | Coagulation factor 5/8 C-terminal domain, discoidin domain (FA58C) |
| OG0000030 | DDE superfamily endonuclease (DDE_SF_endonuclease_dom) |
| OG0000031 | signaling receptor binding |
| OG0000047 | G-protein coupled receptor activity (GPCR) |
| OG0000048 | Domain of unknown function (DUF3504) |
| OG0000063 | ADP-specific glucokinase activity |
| OG0000100 | Ribonuclease H protein |
| OG0000119 | SET (Su(var)3-9, Enhancer-of-zeste, Trithorax) domain |
| OG0000166 | Domain of unknown function (DUF4371) |
| OG0000176 | cobalamin catabolic process |
| OG0000215 | Set1 Ash2 histone methyltransferase complex subunit ASH2 |
| OG0000217 | Unknown function |
| OG0000268 | Unknown function |
| OG0000278 | ShKT domain |
| OG0000302 | positive regulation of bleb assembly |
| OG0000308 | Unknown function |
| OG0000313 | Unknown function |
| OG0000323 | G-protein coupled receptor activity (GPCR) |
| OG0000353 | maintenance of mitotic sister chromatid cohesion, centromeric |
| OG0000358 | Unknown function |
| OG0000368 | MULE transposase domain |
| OG0000405 | Unknown function |
| OG0000408 | Unknown function |
| OG0000424 | Unknown function |
| OG0000435 | Unknown function |
| OG0000448 | Glycosyl transferase family 2 |
| OG0000492 | Choline/Carnitine o-acyltransferase |
| OG0000629 | Protein of unknown function (DUF1280) |
| OG0000647 | SET (Su(var)3-9, Enhancer-of-zeste, Trithorax) domain |
| OG0000673 | K02A2.6-like |
| OG0000695 | ribosomal protein L13 |
| OG0000817 | DNA-directed 5'-3' RNA polymerase activity |
| OG0000875 | DDE superfamily endonuclease |
| OG0000924 | Unknown function |
| OG0001053 | nucleic acid binding |
| OG0001124 | K02A2.6-like |
| OG0001174 | Belongs to the helicase family |
| OG0001179 | cytoskeletal protein binding |
| OG0001620 | Reverse transcriptase (RNA-dependent DNA polymerase) |
| OG0001651 | Unknown function |
| OG0001950 | Unknown function |
| OG0002068 | Unknown function |
| OG0002471 | Unknown function |
| OG0002904 | DEAD/DEAH box helicase |
| OG0003357 | DDE superfamily endonuclease |

**Table S11 GO enrichment results of *Ophiura sarsii vadicola* specific orthogroups.**

| GO ID | Counts | Name | Category | *P*-value |
| --- | --- | --- | --- | --- |
| GO:0007165 | 2 | signal transduction | biological_process | 1.37E-23 |
| GO:0016021 | 4 | integral component of membrane | cellular_component | 3.33E-17 |
| GO:0007283 | 2 | spermatogenesis | biological_process | 5.68E-16 |
| GO:0046872 | 4 | metal ion binding | molecular_function | 8.28E-11 |
| GO:0007601 | 5 | visual perception | biological_process | 3.56E-10 |
| GO:0007155 | 4 | cell adhesion | biological_process | 5.04E-10 |
| GO:0055085 | 2 | transmembrane transport | biological_process | 1.33E-08 |
| GO:0007186 | 7 | G-protein coupled receptor signaling pathway | biological_process | 3.62E-07 |
| GO:0006486 | 4 | protein glycosylation | biological_process | 1.61E-06 |
| GO:0016567 | 3 | protein ubiquitination | biological_process | 3.61E-06 |
| GO:0004930 | 12 | G-protein coupled receptor activity | molecular_function | 4.20E-06 |
| GO:0019233 | 2 | sensory perception of pain | biological_process | 5.74E-06 |
| GO:0033151 | 5 | V(D)J recombination | biological_process | 2.80E-04 |
| GO:0007271 | 2 | synaptic transmission, cholinergic | biological_process | 3.21E-04 |
| GO:0060261 | 7 | positive regulation of transcription initiation from RNA polymerase II promoter | biological_process | 4.37E-04 |
| GO:0004252 | 5 | serine-type endopeptidase activity | molecular_function | 1.31E-03 |
| GO:0006811 | 3 | ion transport | biological_process | 1.32E-03 |
| GO:0005576 | 5 | extracellular region | cellular_component | 1.70E-03 |
| GO:0006468 | 3 | protein phosphorylation | biological_process | 3.63E-03 |
| GO:0002026 | 2 | regulation of the force of heart contraction | biological_process | 4.84E-03 |
| GO:0006694 | 2 | steroid biosynthetic process | biological_process | 1.24E-02 |

**Table S12 GO enrichment results of *Ophiura sarsii* specific orthogroups.**

| GO ID | Counts | Name | Category | *P*-value |
| --- | --- | --- | --- | --- |
| GO:0007165 | 13 | signal transduction | biological_process | 2.45E-72 |
| GO:0016021 | 4 | integral component of membrane | cellular_component | 1.35E-71 |
| GO:0006511 | 6 | ubiquitin-dependent protein catabolic process | biological_process | 2.54E-68 |
| GO:0046872 | 5 | metal ion binding | molecular_function | 2.99E-45 |
| GO:0007601 | 6 | visual perception | biological_process | 7.91E-45 |
| GO:0006805 | 9 | xenobiotic metabolic process | biological_process | 9.93E-44 |
| GO:0006355 | 3 | regulation of transcription, DNA-templated | biological_process | 3.20E-42 |
| GO:0007283 | 17 | spermatogenesis | biological_process | 1.05E-41 |
| GO:0015031 | 4 | protein transport | biological_process | 5.72E-40 |
| GO:0007186 | 7 | G-protein coupled receptor signaling pathway | biological_process | 3.96E-37 |
| GO:0007155 | 9 | cell adhesion | biological_process | 4.54E-37 |
| GO:0007275 | 3 | multicellular organismal development | biological_process | 1.67E-33 |
| GO:0015718 | 12 | monocarboxylic acid transport | biological_process | 5.00E-26 |
| GO:0016567 | 4 | protein ubiquitination | biological_process | 4.97E-25 |
| GO:0004252 | 3 | serine-type endopeptidase activity | molecular_function | 1.52E-20 |
| GO:0042060 | 2 | wound healing | biological_process | 1.29E-19 |
| GO:0006357 | 6 | regulation of transcription from RNA polymerase II promoter | biological_process | 3.41E-19 |
| GO:0005576 | 5 | extracellular region | cellular_component | 1.13E-17 |
| GO:0006811 | 2 | ion transport | biological_process | 6.42E-17 |
| GO:0004930 | 42 | G-protein coupled receptor activity | molecular_function | 1.28E-16 |
| GO:0006313 | 3 | transposition, DNA-mediated | biological_process | 1.42E-15 |
| GO:0006979 | 2 | response to oxidative stress | biological_process | 1.06E-14 |
| GO:0007179 | 2 | transforming growth factor beta receptor signaling pathway | biological_process | 2.27E-10 |
| GO:0019233 | 21 | sensory perception of pain | biological_process | 2.59E-08 |
| GO:0072089 | 3 | stem cell proliferation | biological_process | 8.04E-08 |
| GO:0015074 | 20 | DNA integration | biological_process | 1.12E-07 |
| GO:0043654 | 2 | recognition of apoptotic cell | biological_process | 1.20E-07 |
| GO:0007017 | 4 | microtubule-based process | biological_process | 2.86E-07 |
| GO:0048511 | 3 | rhythmic process | biological_process | 9.77E-07 |
| GO:0002224 | 39 | toll-like receptor signaling pathway | biological_process | 2.23E-06 |
| GO:0050951 | 2 | sensory perception of temperature stimulus | biological_process | 3.18E-06 |
| GO:0006468 | 14 | protein phosphorylation | biological_process | 7.24E-06 |
| GO:0044877 | 3 | macromolecular complex binding | molecular_function | 1.72E-05 |
| GO:0007399 | 10 | nervous system development | biological_process | 1.75E-05 |
| GO:0006898 | 2 | receptor-mediated endocytosis | biological_process | 3.11E-05 |
| GO:0005525 | 3 | GTP binding | molecular_function | 3.28E-05 |
| GO:0004997 | 5 | thyrotropin-releasing hormone receptor activity | molecular_function | 3.41E-05 |
| GO:0097264 | 2 | self proteolysis | biological_process | 3.90E-05 |
| GO:0006887 | 2 | exocytosis | biological_process | 3.90E-05 |
| GO:0006644 | 2 | phospholipid metabolic process | biological_process | 1.48E-04 |
| GO:0070062 | 2 | extracellular exosome | cellular_component | 1.84E-04 |
| GO:0007218 | 6 | neuropeptide signaling pathway | biological_process | 2.28E-04 |
| GO:0051480 | 3 | cytosolic calcium ion homeostasis | biological_process | 2.69E-04 |
| GO:0051403 | 7 | stress-activated MAPK cascade | biological_process | 3.03E-04 |
| GO:0001659 | 2 | temperature homeostasis | biological_process | 6.76E-04 |
| GO:0045907 | 5 | positive regulation of vasoconstriction | biological_process | 7.15E-04 |
| GO:0003779 | 2 | actin binding | molecular_function | 8.37E-04 |
| GO:1990266 | 10 | neutrophil migration | biological_process | 1.05E-03 |
| GO:0070374 | 4 | positive regulation of ERK1 and ERK2 cascade | biological_process | 1.09E-03 |
| GO:0007217 | 6 | tachykinin receptor signaling pathway | biological_process | 1.32E-03 |
| GO:0040008 | 3 | regulation of growth | biological_process | 2.91E-03 |
| GO:1901475 | 3 | pyruvate transmembrane transport | biological_process | 2.91E-03 |
| GO:0042923 | 9 | neuropeptide binding | molecular_function | 2.95E-03 |
| GO:0007266 | 2 | Rho protein signal transduction | biological_process | 4.41E-03 |
| GO:0098908 | 2 | regulation of neuronal action potential | biological_process | 5.39E-03 |
| GO:0051966 | 2 | regulation of synaptic transmission, glutamatergic | biological_process | 6.58E-03 |
| GO:0017015 | 6 | regulation of transforming growth factor beta receptor signaling pathway | biological_process | 6.63E-03 |
| GO:0042981 | 3 | regulation of apoptotic process | biological_process | 8.86E-03 |
| GO:0009725 | 3 | response to hormone | biological_process | 8.86E-03 |
| GO:0051928 | 5 | positive regulation of calcium ion transport | biological_process | 9.18E-03 |
| GO:0006310 | 4 | DNA recombination | biological_process | 9.19E-03 |
| GO:0050773 | 2 | regulation of dendrite development | biological_process | 9.75E-03 |
| GO:0019216 | 2 | regulation of lipid metabolic process | biological_process | 9.75E-03 |
| GO:0051259 | 4 | protein oligomerization | biological_process | 1.50E-02 |
| GO:0006334 | 48 | nucleosome assembly | biological_process | 1.50E-02 |
| GO:0034340 | 13 | response to type I interferon | biological_process | 1.64E-02 |
| GO:0032197 | 5 | transposition, RNA-mediated | biological_process | 1.96E-02 |
| GO:0035666 | 9 | TRIF-dependent toll-like receptor signaling pathway | biological_process | 2.01E-02 |
| GO:0048265 | 17 | response to pain | biological_process | 2.02E-02 |
| GO:0006096 | 2 | glycolytic process | biological_process | 2.09E-02 |
| GO:0009658 | 9 | chloroplast organization | biological_process | 2.36E-02 |
| GO:0019441 | 3 | tryptophan catabolic process to kynurenine | biological_process | 2.50E-02 |
| GO:0006807 | 3 | nitrogen compound metabolic process | biological_process | 2.50E-02 |
| GO:0046982 | 41 | protein heterodimerization activity | molecular_function | 2.79E-02 |

**Table S13** **GO enrichment results of *Amphiura filiformis* specific orthogroups.**

| GO ID | Counts | Name | Category | *P*-value |
| --- | --- | --- | --- | --- |
| GO:0046872 | 42 | metal ion binding | molecular_function | 0.00 |
| GO:0006805 | 66 | xenobiotic metabolic process | biological_process | 0.00 |
| GO:0007601 | 45 | visual perception | biological_process | 0.00 |
| GO:0006355 | 26 | regulation of transcription, DNA-templated | biological_process | 0.00 |
| GO:0016021 | 15 | integral component of membrane | cellular_component | 0.00 |
| GO:0007165 | 72 | signal transduction | biological_process | 0.00 |
| GO:0004930 | 15 | G-protein coupled receptor activity | molecular_function | 0.00 |
| GO:0015031 | 14 | protein transport | biological_process | 0.00 |
| GO:0007283 | 24 | spermatogenesis | biological_process | 0.00 |
| GO:0006511 | 31 | ubiquitin-dependent protein catabolic process | biological_process | 0.00 |
| GO:0007155 | 50 | cell adhesion | biological_process | 3.36E-322 |
| GO:0007186 | 45 | G-protein coupled receptor signaling pathway | biological_process | 1.20E-307 |
| GO:0055085 | 13 | transmembrane transport | biological_process | 4.82E-277 |
| GO:0016032 | 27 | viral process | biological_process | 5.53E-271 |
| GO:0015718 | 42 | monocarboxylic acid transport | biological_process | 4.04E-254 |
| GO:0016055 | 8 | Wnt signaling pathway | biological_process | 4.23E-253 |
| GO:0016192 | 3 | vesicle-mediated transport | biological_process | 4.27E-240 |
| GO:0006486 | 26 | protein glycosylation | biological_process | 1.79E-233 |
| GO:0006508 | 26 | proteolysis | biological_process | 4.41E-229 |
| GO:0007275 | 40 | multicellular organismal development | biological_process | 4.95E-229 |
| GO:0008270 | 8 | zinc ion binding | molecular_function | 2.67E-208 |
| GO:0019233 | 4 | sensory perception of pain | biological_process | 6.68E-205 |
| GO:0008380 | 5 | RNA splicing | biological_process | 3.18E-202 |
| GO:0016567 | 22 | protein ubiquitination | biological_process | 5.06E-200 |
| GO:0004252 | 16 | serine-type endopeptidase activity | molecular_function | 1.56E-160 |
| GO:0005576 | 18 | extracellular region | cellular_component | 1.42E-151 |
| GO:0046982 | 26 | protein heterodimerization activity | molecular_function | 4.75E-145 |
| GO:0005509 | 10 | calcium ion binding | molecular_function | 4.83E-136 |
| GO:0006979 | 2 | response to oxidative stress | biological_process | 8.37E-130 |
| GO:0006468 | 3 | protein phosphorylation | biological_process | 4.13E-129 |
| GO:0006313 | 14 | transposition, DNA-mediated | biological_process | 3.16E-121 |
| GO:0006811 | 15 | ion transport | biological_process | 3.21E-120 |
| GO:0007338 | 45 | single fertilization | biological_process | 7.94E-118 |
| GO:0042060 | 29 | wound healing | biological_process | 2.68E-117 |
| GO:0016491 | 3 | oxidoreductase activity | molecular_function | 7.78E-115 |
| GO:0006814 | 12 | sodium ion transport | biological_process | 4.10E-112 |
| GO:0015074 | 48 | DNA integration | biological_process | 3.24E-106 |
| GO:0007169 | 27 | transmembrane receptor protein tyrosine kinase signaling pathway | biological_process | 2.23E-104 |
| GO:0007605 | 23 | sensory perception of sound | biological_process | 2.45E-100 |
| GO:0007166 | 15 | cell surface receptor signaling pathway | biological_process | 3.37E-98 |
| GO:0009617 | 12 | response to bacterium | biological_process | 1.19E-95 |
| GO:0007018 | 12 | microtubule-based movement | biological_process | 7.59E-94 |
| GO:0005524 | 9 | ATP binding | molecular_function | 1.66E-91 |
| GO:0030433 | 2 | ER-associated ubiquitin-dependent protein catabolic process | biological_process | 2.88E-91 |
| GO:0007399 | 7 | nervous system development | biological_process | 5.15E-91 |
| GO:0000398 | 2 | mRNA splicing, via spliceosome | biological_process | 8.94E-86 |
| GO:0006470 | 11 | protein dephosphorylation | biological_process | 3.33E-83 |
| GO:0006694 | 7 | steroid biosynthetic process | biological_process | 2.50E-79 |
| GO:0006334 | 41 | nucleosome assembly | biological_process | 7.12E-79 |
| GO:0007017 | 4 | microtubule-based process | biological_process | 1.26E-76 |
| GO:0005886 | 12 | plasma membrane | cellular_component | 3.15E-75 |
| GO:0007271 | 43 | synaptic transmission, cholinergic | biological_process | 1.70E-74 |
| GO:0007179 | 11 | transforming growth factor beta receptor signaling pathway | biological_process | 1.58E-73 |
| GO:0006790 | 2 | sulfur compound metabolic process | biological_process | 3.33E-72 |
| GO:0042572 | 10 | retinol metabolic process | biological_process | 3.08E-69 |
| GO:0001570 | 4 | vasculogenesis | biological_process | 1.01E-67 |
| GO:0004674 | 27 | protein serine/threonine kinase activity | molecular_function | 2.07E-67 |
| GO:0006457 | 8 | protein folding | biological_process | 8.00E-67 |
| GO:0007416 | 8 | synapse assembly | biological_process | 5.93E-66 |
| GO:0006413 | 2 | translational initiation | biological_process | 6.00E-66 |
| GO:0042923 | 3 | neuropeptide binding | molecular_function | 1.87E-65 |
| GO:0060012 | 2 | synaptic transmission, glycinergic | biological_process | 4.65E-65 |
| GO:0008542 | 7 | visual learning | biological_process | 2.35E-64 |
| GO:0006351 | 5 | transcription, DNA-templated | biological_process | 6.79E-64 |
| GO:0043252 | 14 | sodium-independent organic anion transport | biological_process | 7.86E-60 |
| GO:0006631 | 35 | fatty acid metabolic process | biological_process | 5.67E-58 |
| GO:0016020 | 18 | membrane | cellular_component | 4.61E-56 |
| GO:0072089 | 14 | stem cell proliferation | biological_process | 1.19E-55 |
| GO:0051403 | 9 | stress-activated MAPK cascade | biological_process | 1.48E-55 |
| GO:0007194 | 15 | negative regulation of adenylate cyclase activity | biological_process | 4.49E-54 |
| GO:0007286 | 4 | spermatid development | biological_process | 7.64E-53 |
| GO:0045087 | 19 | innate immune response | biological_process | 3.96E-52 |
| GO:0009636 | 10 | response to toxic substance | biological_process | 2.56E-51 |
| GO:0043523 | 11 | regulation of neuron apoptotic process | biological_process | 1.69E-50 |
| GO:0006665 | 2 | sphingolipid metabolic process | biological_process | 4.30E-50 |
| GO:0006281 | 2 | DNA repair | biological_process | 4.30E-50 |
| GO:0072553 | 2 | terminal button organization | biological_process | 4.30E-50 |
| GO:0050951 | 6 | sensory perception of temperature stimulus | biological_process | 3.63E-48 |
| GO:0006417 | 3 | regulation of translation | biological_process | 5.84E-48 |
| GO:0009268 | 4 | response to pH | biological_process | 5.83E-46 |
| GO:0031638 | 17 | zymogen activation | biological_process | 1.39E-44 |
| GO:0002224 | 18 | toll-like receptor signaling pathway | biological_process | 5.90E-44 |
| GO:0009658 | 7 | chloroplast organization | biological_process | 7.31E-44 |
| GO:0043654 | 16 | recognition of apoptotic cell | biological_process | 1.24E-43 |
| GO:0034340 | 20 | response to type I interferon | biological_process | 1.56E-43 |
| GO:0017015 | 4 | regulation of transforming growth factor beta receptor signaling pathway | biological_process | 2.11E-43 |
| GO:0070374 | 4 | positive regulation of ERK1 and ERK2 cascade | biological_process | 1.06E-41 |
| GO:0005525 | 9 | GTP binding | molecular_function | 2.39E-41 |
| GO:0097225 | 4 | sperm midpiece | cellular_component | 7.46E-41 |
| GO:0006898 | 6 | receptor-mediated endocytosis | biological_process | 9.42E-40 |
| GO:0051923 | 8 | sulfation | biological_process | 7.25E-39 |
| GO:0070212 | 6 | protein poly-ADP-ribosylation | biological_process | 4.35E-38 |
| GO:0070207 | 9 | protein homotrimerization | biological_process | 4.48E-38 |
| GO:0030246 | 26 | carbohydrate binding | molecular_function | 5.30E-38 |
| GO:0006493 | 2 | protein O-linked glycosylation | biological_process | 5.34E-38 |
| GO:0055114 | 4 | oxidation-reduction process | biological_process | 1.78E-37 |
| GO:0043312 | 4 | neutrophil degranulation | biological_process | 8.52E-36 |
| GO:0046208 | 7 | spermine catabolic process | biological_process | 1.39E-35 |
| GO:0006986 | 3 | response to unfolded protein | biological_process | 3.79E-35 |
| GO:0016042 | 8 | lipid catabolic process | biological_process | 8.77E-35 |
| GO:0031146 | 2 | SCF-dependent proteasomal ubiquitin-dependent protein catabolic process | biological_process | 1.39E-34 |
| GO:0097264 | 9 | self proteolysis | biological_process | 4.98E-34 |
| GO:0046039 | 5 | GTP metabolic process | biological_process | 5.18E-34 |
| GO:0016126 | 14 | sterol biosynthetic process | biological_process | 7.46E-34 |
| GO:1901475 | 3 | pyruvate transmembrane transport | biological_process | 1.84E-33 |
| GO:0040008 | 3 | regulation of growth | biological_process | 1.84E-33 |
| GO:0021591 | 10 | ventricular system development | biological_process | 2.59E-33 |
| GO:0035725 | 8 | sodium ion transmembrane transport | biological_process | 2.34E-32 |
| GO:0007214 | 6 | gamma-aminobutyric acid signaling pathway | biological_process | 2.56E-32 |
| GO:0006904 | 3 | vesicle docking involved in exocytosis | biological_process | 6.06E-31 |
| GO:0032197 | 5 | transposition, RNA-mediated | biological_process | 1.03E-30 |
| GO:0042981 | 2 | regulation of apoptotic process | biological_process | 2.41E-30 |
| GO:0050982 | 3 | detection of mechanical stimulus | biological_process | 4.16E-30 |
| GO:0048010 | 3 | vascular endothelial growth factor receptor signaling pathway | biological_process | 2.84E-29 |
| GO:0015804 | 9 | neutral amino acid transport | biological_process | 3.04E-29 |
| GO:0006909 | 12 | phagocytosis | biological_process | 7.18E-29 |
| GO:0044877 | 20 | macromolecular complex binding | molecular_function | 2.47E-28 |
| GO:0006887 | 13 | exocytosis | biological_process | 2.82E-28 |
| GO:0001507 | 5 | acetylcholine catabolic process in synaptic cleft | biological_process | 2.94E-28 |
| GO:0022857 | 3 | transmembrane transporter activity | molecular_function | 1.31E-27 |
| GO:0004518 | 5 | nuclease activity | molecular_function | 1.92E-27 |
| GO:0014048 | 11 | regulation of glutamate secretion | biological_process | 3.64E-27 |
| GO:0044319 | 2 | wound healing, spreading of cells | biological_process | 5.60E-27 |
| GO:0035556 | 2 | intracellular signal transduction | biological_process | 5.60E-27 |
| GO:0019432 | 3 | triglyceride biosynthetic process | biological_process | 8.87E-27 |
| GO:0006310 | 7 | DNA recombination | biological_process | 1.08E-26 |
| GO:0019319 | 4 | hexose biosynthetic process | biological_process | 1.13E-26 |
| GO:0060041 | 2 | retina development in camera-type eye | biological_process | 3.86E-26 |
| GO:0032496 | 2 | response to lipopolysaccharide | biological_process | 3.86E-26 |
| GO:0048771 | 5 | tissue remodeling | biological_process | 8.02E-26 |
| GO:0007596 | 15 | blood coagulation | biological_process | 1.15E-25 |
| GO:0050821 | 2 | protein stabilization | biological_process | 2.65E-25 |
| GO:0016705 | 2 | oxidoreductase activity, acting on paired donors, with incorporation or reduction of molecular oxygen | molecular_function | 2.65E-25 |
| GO:0050852 | 7 | T cell receptor signaling pathway | biological_process | 4.18E-25 |
| GO:0006954 | 5 | inflammatory response | biological_process | 5.15E-25 |
| GO:0045329 | 11 | carnitine biosynthetic process | biological_process | 7.29E-25 |
| GO:0051770 | 7 | positive regulation of nitric-oxide synthase biosynthetic process | biological_process | 2.57E-24 |
| GO:0008484 | 7 | sulfuric ester hydrolase activity | molecular_function | 2.57E-24 |
| GO:0006693 | 3 | prostaglandin metabolic process | biological_process | 2.67E-24 |
| GO:0051966 | 3 | regulation of synaptic transmission, glutamatergic | biological_process | 2.67E-24 |
| GO:0000038 | 5 | very long-chain fatty acid metabolic process | biological_process | 3.29E-24 |
| GO:0015020 | 5 | glucuronosyltransferase activity | molecular_function | 3.29E-24 |
| GO:0071577 | 15 | zinc II ion transmembrane transport | biological_process | 3.47E-24 |
| GO:0015881 | 3 | creatine transport | biological_process | 1.77E-23 |
| GO:0070904 | 15 | transepithelial L-ascorbic acid transport | biological_process | 1.89E-23 |
| GO:0016614 | 15 | oxidoreductase activity, acting on CH-OH group of donors | molecular_function | 1.89E-23 |
| GO:0003779 | 9 | actin binding | molecular_function | 5.28E-23 |
| GO:0006897 | 3 | endocytosis | biological_process | 1.17E-22 |
| GO:0035249 | 3 | synaptic transmission, glutamatergic | biological_process | 1.17E-22 |
| GO:0003964 | 5 | RNA-directed DNA polymerase activity | molecular_function | 1.32E-22 |
| GO:0060879 | 6 | semicircular canal fusion | biological_process | 7.22E-22 |
| GO:0001666 | 2 | response to hypoxia | biological_process | 3.83E-21 |
| GO:0042135 | 6 | neurotransmitter catabolic process | biological_process | 4.42E-21 |
| GO:0007160 | 3 | cell-matrix adhesion | biological_process | 5.06E-21 |
| GO:0016322 | 7 | neuron remodeling | biological_process | 2.07E-20 |
| GO:0050655 | 2 | dermatan sulfate proteoglycan metabolic process | biological_process | 2.57E-20 |
| GO:0005506 | 5 | iron ion binding | molecular_function | 3.22E-20 |
| GO:0019441 | 5 | tryptophan catabolic process to kynurenine | biological_process | 3.22E-20 |
| GO:0006641 | 3 | triglyceride metabolic process | biological_process | 3.30E-20 |
| GO:0010842 | 4 | retina layer formation | biological_process | 3.49E-20 |
| GO:0009115 | 4 | xanthine catabolic process | biological_process | 3.49E-20 |
| GO:0042359 | 2 | vitamin D metabolic process | biological_process | 1.71E-19 |
| GO:0008654 | 2 | phospholipid biosynthetic process | biological_process | 1.71E-19 |
| GO:0043687 | 3 | post-translational protein modification | biological_process | 2.14E-19 |
| GO:0050807 | 4 | regulation of synapse organization | biological_process | 2.20E-19 |
| GO:0018095 | 13 | protein polyglutamylation | biological_process | 2.47E-19 |
| GO:0043065 | 2 | positive regulation of apoptotic process | biological_process | 1.14E-18 |
| GO:0050808 | 2 | synapse organization | biological_process | 1.14E-18 |
| GO:0031175 | 2 | neuron projection development | biological_process | 1.14E-18 |
| GO:0046274 | 2 | lignin catabolic process | biological_process | 1.14E-18 |
| GO:0065003 | 5 | macromolecular complex assembly | biological_process | 1.21E-18 |
| GO:0010811 | 3 | positive regulation of cell-substrate adhesion | biological_process | 1.38E-18 |
| GO:0009408 | 3 | response to heat | biological_process | 1.38E-18 |
| GO:0016485 | 5 | protein processing | biological_process | 7.37E-18 |
| GO:0051604 | 2 | protein maturation | biological_process | 7.54E-18 |
| GO:0010033 | 2 | response to organic substance | biological_process | 7.54E-18 |
| GO:0007130 | 2 | synaptonemal complex assembly | biological_process | 7.54E-18 |
| GO:0006833 | 2 | water transport | biological_process | 7.54E-18 |
| GO:0035456 | 3 | response to interferon-beta | biological_process | 8.82E-18 |
| GO:0060071 | 6 | Wnt signaling pathway, planar cell polarity pathway | biological_process | 3.38E-17 |
| GO:0003858 | 3 | 3-hydroxybutyrate dehydrogenase activity | molecular_function | 5.62E-17 |
| GO:0005328 | 3 | neurotransmitter:sodium symporter activity | molecular_function | 5.62E-17 |
| GO:0034243 | 5 | regulation of transcription elongation from RNA polymerase II promoter | biological_process | 2.65E-16 |
| GO:0019285 | 2 | glycine betaine biosynthetic process from choline | biological_process | 3.25E-16 |
| GO:0050896 | 2 | response to stimulus | biological_process | 3.25E-16 |
| GO:0001649 | 3 | osteoblast differentiation | biological_process | 3.56E-16 |
| GO:0097374 | 3 | sensory neuron axon guidance | biological_process | 3.56E-16 |
| GO:0043588 | 4 | skin development | biological_process | 1.99E-15 |
| GO:0030435 | 2 | sporulation resulting in formation of a cellular spore | biological_process | 2.11E-15 |
| GO:0042554 | 2 | superoxide anion generation | biological_process | 2.11E-15 |
| GO:0006953 | 15 | acute-phase response | biological_process | 6.00E-15 |
| GO:0045214 | 17 | sarcomere organization | biological_process | 7.36E-15 |
| GO:0042246 | 5 | tissue regeneration | biological_process | 9.23E-15 |
| GO:0005212 | 5 | structural constituent of eye lens | molecular_function | 9.23E-15 |
| GO:0006874 | 5 | cellular calcium ion homeostasis | biological_process | 9.23E-15 |
| GO:0006572 | 5 | tyrosine catabolic process | biological_process | 9.23E-15 |
| GO:0009314 | 2 | response to radiation | biological_process | 1.37E-14 |
| GO:2000300 | 2 | regulation of synaptic vesicle exocytosis | biological_process | 1.37E-14 |
| GO:0010043 | 2 | response to zinc ion | biological_process | 1.37E-14 |
| GO:0048538 | 2 | thymus development | biological_process | 1.37E-14 |
| GO:0008146 | 3 | sulfotransferase activity | molecular_function | 1.39E-14 |
| GO:0019236 | 6 | response to pheromone | biological_process | 3.71E-14 |
| GO:0050770 | 19 | regulation of axonogenesis | biological_process | 3.75E-14 |
| GO:0006542 | 3 | glutamine biosynthetic process | biological_process | 8.62E-14 |
| GO:0002040 | 2 | sprouting angiogenesis | biological_process | 8.79E-14 |
| GO:0016747 | 2 | transferase activity, transferring acyl groups other than amino-acyl groups | molecular_function | 8.79E-14 |
| GO:0009166 | 23 | nucleotide catabolic process | biological_process | 1.65E-13 |
| GO:0048471 | 5 | perinuclear region of cytoplasm | cellular_component | 3.09E-13 |
| GO:0001964 | 5 | startle response | biological_process | 3.09E-13 |
| GO:0035666 | 3 | TRIF-dependent toll-like receptor signaling pathway | biological_process | 5.28E-13 |
| GO:0052695 | 3 | cellular glucuronidation | biological_process | 5.28E-13 |
| GO:0016125 | 2 | sterol metabolic process | biological_process | 5.61E-13 |
| GO:0009650 | 2 | UV protection | biological_process | 5.61E-13 |
| GO:0007417 | 9 | central nervous system development | biological_process | 1.26E-12 |
| GO:0006766 | 8 | vitamin metabolic process | biological_process | 2.23E-12 |
| GO:0002223 | 4 | stimulatory C-type lectin receptor signaling pathway | biological_process | 2.50E-12 |
| GO:0004888 | 4 | transmembrane signaling receptor activity | molecular_function | 2.50E-12 |
| GO:0010025 | 4 | wax biosynthetic process | biological_process | 2.50E-12 |
| GO:0009313 | 17 | oligosaccharide catabolic process | biological_process | 3.02E-12 |
| GO:0001878 | 2 | response to yeast | biological_process | 3.55E-12 |
| GO:0006401 | 4 | RNA catabolic process | biological_process | 1.45E-11 |
| GO:0060348 | 10 | bone development | biological_process | 1.70E-11 |
| GO:0006941 | 3 | striated muscle contraction | biological_process | 1.93E-11 |
| GO:0043066 | 3 | negative regulation of apoptotic process | biological_process | 1.93E-11 |
| GO:0050955 | 3 | thermoception | biological_process | 1.93E-11 |
| GO:0003746 | 2 | translation elongation factor activity | molecular_function | 2.23E-11 |
| GO:0032012 | 2 | regulation of ARF protein signal transduction | biological_process | 2.23E-11 |
| GO:0001944 | 5 | vasculature development | biological_process | 5.47E-11 |
| GO:0060707 | 4 | trophoblast giant cell differentiation | biological_process | 8.29E-11 |
| GO:0003223 | 3 | ventricular compact myocardium morphogenesis | biological_process | 1.15E-10 |
| GO:0042802 | 2 | identical protein binding | molecular_function | 1.39E-10 |
| GO:0007156 | 3 | homophilic cell adhesion via plasma membrane adhesion molecules | biological_process | 6.75E-10 |
| GO:0050750 | 3 | low-density lipoprotein particle receptor binding | molecular_function | 6.75E-10 |
| GO:0042110 | 2 | T cell activation | biological_process | 8.56E-10 |
| GO:0035914 | 2 | skeletal muscle cell differentiation | biological_process | 8.56E-10 |
| GO:0043052 | 3 | thermotaxis | biological_process | 3.92E-09 |
| GO:0030163 | 3 | protein catabolic process | biological_process | 3.92E-09 |
| GO:0033197 | 3 | response to vitamin E | biological_process | 3.92E-09 |
| GO:0035372 | 2 | protein localization to microtubule | biological_process | 5.21E-09 |
| GO:0009253 | 2 | peptidoglycan catabolic process | biological_process | 5.21E-09 |
| GO:0090244 | 2 | Wnt signaling pathway involved in somitogenesis | biological_process | 5.21E-09 |
| GO:0019530 | 2 | taurine metabolic process | biological_process | 5.21E-09 |
| GO:0035313 | 2 | wound healing, spreading of epidermal cells | biological_process | 5.21E-09 |
| GO:0035774 | 2 | positive regulation of insulin secretion involved in cellular response to glucose stimulus | biological_process | 5.21E-09 |
| GO:0052689 | 8 | carboxylic ester hydrolase activity | molecular_function | 6.84E-09 |
| GO:0009749 | 5 | response to glucose | biological_process | 8.50E-09 |
| GO:0060261 | 7 | positive regulation of transcription initiation from RNA polymerase II promoter | biological_process | 1.30E-08 |
| GO:0032259 | 13 | methylation | biological_process | 1.79E-08 |
| GO:0004867 | 3 | serine-type endopeptidase inhibitor activity | molecular_function | 2.24E-08 |
| GO:0098609 | 3 | cell-cell adhesion | biological_process | 2.24E-08 |
| GO:0019226 | 3 | transmission of nerve impulse | biological_process | 2.24E-08 |
| GO:0040011 | 3 | locomotion | biological_process | 2.24E-08 |
| GO:0015734 | 3 | taurine transport | biological_process | 2.24E-08 |
| GO:0046686 | 2 | response to cadmium ion | biological_process | 3.14E-08 |
| GO:0051693 | 2 | actin filament capping | biological_process | 3.14E-08 |
| GO:0034314 | 2 | Arp2/3 complex-mediated actin nucleation | biological_process | 3.14E-08 |
| GO:0005044 | 11 | scavenger receptor activity | molecular_function | 7.67E-08 |
| GO:0004104 | 6 | cholinesterase activity | molecular_function | 1.20E-07 |
| GO:0019628 | 6 | urate catabolic process | biological_process | 1.20E-07 |
| GO:0007185 | 3 | transmembrane receptor protein tyrosine phosphatase signaling pathway | biological_process | 1.25E-07 |
| GO:0034612 | 3 | response to tumor necrosis factor | biological_process | 1.25E-07 |
| GO:0036462 | 3 | TRAIL-activated apoptotic signaling pathway | biological_process | 1.25E-07 |
| GO:0016874 | 2 | ligase activity | molecular_function | 1.86E-07 |
| GO:0031408 | 2 | oxylipin biosynthetic process | biological_process | 1.86E-07 |
| GO:0070206 | 2 | protein trimerization | biological_process | 1.86E-07 |
| GO:0008064 | 2 | regulation of actin polymerization or depolymerization | biological_process | 1.86E-07 |
| GO:0061400 | 2 | positive regulation of transcription from RNA polymerase II promoter in response to calcium ion | biological_process | 1.86E-07 |
| GO:0009744 | 2 | response to sucrose | biological_process | 1.86E-07 |
| GO:0010344 | 7 | seed oilbody biogenesis | biological_process | 2.94E-07 |
| GO:0051262 | 2 | protein tetramerization | biological_process | 1.08E-06 |
| GO:0043129 | 2 | surfactant homeostasis | biological_process | 1.08E-06 |
| GO:0021559 | 2 | trigeminal nerve development | biological_process | 1.08E-06 |
| GO:0051287 | 2 | NAD binding | molecular_function | 1.08E-06 |
| GO:0018094 | 4 | protein polyglycylation | biological_process | 2.07E-06 |
| GO:0030414 | 4 | peptidase inhibitor activity | molecular_function | 2.07E-06 |
| GO:0008152 | 4 | metabolic process | biological_process | 2.07E-06 |
| GO:0006703 | 10 | estrogen biosynthetic process | biological_process | 2.73E-06 |
| GO:0034138 | 3 | toll-like receptor 3 signaling pathway | biological_process | 3.71E-06 |
| GO:0050915 | 3 | sensory perception of sour taste | biological_process | 3.71E-06 |
| GO:0052697 | 3 | xenobiotic glucuronidation | biological_process | 3.71E-06 |
| GO:0006297 | 3 | nucleotide-excision repair, DNA gap filling | biological_process | 3.71E-06 |
| GO:0010756 | 2 | positive regulation of plasminogen activation | biological_process | 6.19E-06 |
| GO:0003429 | 2 | growth plate cartilage chondrocyte morphogenesis | biological_process | 6.19E-06 |
| GO:1902947 | 2 | regulation of tau-protein kinase activity | biological_process | 6.19E-06 |
| GO:0007614 | 2 | short-term memory | biological_process | 6.19E-06 |
| GO:0097494 | 2 | regulation of vesicle size | biological_process | 6.19E-06 |
| GO:0017134 | 2 | fibroblast growth factor binding | molecular_function | 6.19E-06 |
| GO:0019346 | 2 | transsulfuration | biological_process | 6.19E-06 |
| GO:0021517 | 2 | ventral spinal cord development | biological_process | 6.19E-06 |
| GO:0021510 | 10 | spinal cord development | biological_process | 1.08E-05 |
| GO:0030168 | 6 | platelet activation | biological_process | 1.22E-05 |
| GO:0034453 | 3 | microtubule anchoring | biological_process | 1.94E-05 |
| GO:0039529 | 3 | RIG-I signaling pathway | biological_process | 1.94E-05 |
| GO:0010469 | 5 | regulation of receptor activity | biological_process | 2.48E-05 |
| GO:0005993 | 2 | trehalose catabolic process | biological_process | 3.45E-05 |
| GO:0046903 | 2 | secretion | biological_process | 3.45E-05 |
| GO:0015739 | 2 | sialic acid transport | biological_process | 3.45E-05 |
| GO:0051969 | 4 | regulation of transmission of nerve impulse | biological_process | 5.00E-05 |
| GO:0005272 | 3 | sodium channel activity | molecular_function | 9.85E-05 |
| GO:0006013 | 3 | mannose metabolic process | biological_process | 9.85E-05 |
| GO:0008218 | 5 | bioluminescence | biological_process | 1.11E-04 |
| GO:0007367 | 2 | segment polarity determination | biological_process | 1.87E-04 |
| GO:0010044 | 2 | response to aluminum ion | biological_process | 1.87E-04 |
| GO:0009733 | 2 | response to auxin | biological_process | 1.87E-04 |
| GO:0003044 | 2 | regulation of systemic arterial blood pressure mediated by a chemical signal | biological_process | 9.73E-04 |
| GO:0070086 | 2 | ubiquitin-dependent endocytosis | biological_process | 9.73E-04 |
| GO:0001895 | 2 | retina homeostasis | biological_process | 9.73E-04 |
| GO:0060074 | 2 | synapse maturation | biological_process | 9.73E-04 |
| GO:0048814 | 2 | regulation of dendrite morphogenesis | biological_process | 9.73E-04 |
| GO:0032483 | 2 | regulation of Rab protein signal transduction | biological_process | 9.73E-04 |
| GO:0007522 | 2 | visceral muscle development | biological_process | 9.73E-04 |
| GO:0015786 | 2 | UDP-glucose transport | biological_process | 9.73E-04 |
| GO:0021649 | 4 | vestibulocochlear nerve structural organization | biological_process | 1.03E-03 |
| GO:0006605 | 4 | protein targeting | biological_process | 1.03E-03 |
| GO:0007173 | 3 | epidermal growth factor receptor signaling pathway | biological_process | 2.22E-03 |
| GO:0007009 | 3 | plasma membrane organization | biological_process | 2.22E-03 |
| GO:0046135 | 3 | pyrimidine nucleoside catabolic process | biological_process | 2.22E-03 |
| GO:0034134 | 11 | toll-like receptor 2 signaling pathway | biological_process | 2.58E-03 |
| GO:0034142 | 4 | toll-like receptor 4 signaling pathway | biological_process | 4.28E-03 |
| GO:0005991 | 4 | trehalose metabolic process | biological_process | 4.28E-03 |
| GO:0007610 | 4 | behavior | biological_process | 4.28E-03 |
| GO:2000484 | 2 | positive regulation of interleukin-8 secretion | biological_process | 4.82E-03 |
| GO:0034341 | 2 | response to interferon-gamma | biological_process | 4.82E-03 |
| GO:0006654 | 2 | phosphatidic acid biosynthetic process | biological_process | 4.82E-03 |
| GO:0004222 | 2 | metalloendopeptidase activity | molecular_function | 4.82E-03 |
| GO:0017186 | 2 | peptidyl-pyroglutamic acid biosynthetic process, using glutaminyl-peptide cyclotransferase | biological_process | 4.82E-03 |
| GO:0002576 | 5 | platelet degranulation | biological_process | 7.32E-03 |
| GO:0006590 | 5 | thyroid hormone generation | biological_process | 7.32E-03 |
| GO:0051124 | 3 | synaptic growth at neuromuscular junction | biological_process | 9.63E-03 |
| GO:1903775 | 3 | regulation of DNA double-strand break processing | biological_process | 9.63E-03 |
| GO:0030245 | 6 | cellulose catabolic process | biological_process | 1.15E-02 |
| GO:0051607 | 2 | defense response to virus | biological_process | 2.22E-02 |
| GO:0032571 | 2 | response to vitamin K | biological_process | 2.22E-02 |
| GO:0033292 | 2 | T-tubule organization | biological_process | 2.22E-02 |
| GO:0015012 | 2 | heparan sulfate proteoglycan biosynthetic process | biological_process | 2.22E-02 |
| GO:0043254 | 2 | regulation of protein complex assembly | biological_process | 2.22E-02 |
| GO:0051091 | 5 | positive regulation of sequence-specific DNA binding transcription factor activity | biological_process | 2.54E-02 |
| GO:0050909 | 5 | sensory perception of taste | biological_process | 2.54E-02 |
| GO:0002456 | 3 | T cell mediated immunity | biological_process | 3.81E-02 |
| GO:0006740 | 3 | NADPH regeneration | biological_process | 3.81E-02 |
| GO:0018917 | 3 | fluorene metabolic process | biological_process | 3.81E-02 |

**Table S14 Significantly differentially expressed key genes during regeneration using RNA sequencing data.**

| Group comparison | Gene ID | Up/Down | Gene | Annotation |
| --- | --- | --- | --- | --- |
| D13 vs D1 | evm.TU.CTG.2000.12 | Down | *BTG3* | Promote cell cycle progression from G0/G1 phase to S phase |
|  | evm.TU.CTG.2231.68 | Down | *MAP3K20* | Promote apoptosis |
|  | evm.TU.CTG.4186.36 | Down | *MUC2* | The mucosal surface barrier prevents infection |
|  | evm.TU.CTG.3813.1 | Down | *Snx13* | Maintain nuclear homeostasis |
|  | evm.TU.CTG.1918.1 | Down | *MBNL1* | Highly expressed during differentiation of heart, skeletal muscle and myoblasts |
|  | evm.TU.CTG.1686.12 | Up | *lin-28* | It controls the number of cells during development |
|  | evm.TU.CTG.750.7 | Up | *Titin* | Embryonic striated muscle and muscle tendon are key components of assembly function ([Zhang et al., 2000](#_ENREF_38)) |
|  | evm.TU.CTG.1093.112 | Up | *DMBT1* | It plays a role in mucosal defense, cellular immune defense and epithelial differentiation |
|  | evm.TU.CTG.3787.30 | Down | *SLC8A3* | Glial cell differentiation is associated with normal myelination |
| D13 vs D3 | evm.TU.CTG.3409.10 | Down | *CSRP2* | Promote smooth muscle cell proliferation and dedifferentiation, and play a role in the development of embryonic vascular system |
|  | evm.TU.CTG.1319.2 | Down | *DLG5* | Involved in epithelial-mesenchymal transition (EMT). Plays an important role in the formation of neurons and synapses |
|  | evm.TU.CTG.1378.38 | Down | *MAP-1B* | Highly expressed during nervous system development |
|  | evm.TU.CTG.3911.13 | Down | *Ap-cadherin* | Calcium-dependent cell adhesion proteins |
|  | evm.TU.CTG.689.100 | Up | *Muc4* | Inhibit cell apoptosis and stimulate cell proliferation. |
|  | evm.TU.CTG.1484.82 | Up | *HTR4* | It is mediated by a G protein as a mitogen |
|  | evm.TU.CTG.2102.66 | Up | *COL8A1* | It plays an important role in the migration and proliferation of smooth muscle cells |
|  | evm.TU.CTG.2591.28 | Up | *BP10* | Possibly involved in differentiation of ectodermal lineages and subsequent embryonic development |
|  | evm.TU.CTG.2025.10 | Down | *SIK3* | Involved in the regulation of mTOR signaling in chondrocyte differentiation during bone formation |
| D13 vs D7 | evm.TU.CTG.4209.8 | Down | *FAM13A* | GPCR signal transduction |
|  | evm.TU.CTG.1378.38 | Down | *MAP-1B* | It is highly expressed during nervous system development |
|  | evm.TU.CTG.3554.8 | Down | *Ski* | It plays a role in the terminal differentiation of skeletal muscle cells |
|  | evm.TU.CTG.417.32 | Up | *Twitchin/unc22* | It plays a role in the musculature of the body wall |
|  | evm.TU.CTG.4097.55 | Up | *SPEN* | It can inhibit different critical pathways, such as Notch |
|  | evm.TU.CTG.1318.50 | Up | *RPS15A* | 40S ribosomal protein S15a |
|  | evm.TU.CTG.140.7 | Up | *Rplp2* | 60S acidic ribosomal protein P2 |
|  | evm.TU.CTG.2348.77 | Up | *RpL37a* | Probable 60S ribosomal protein L37-A |

**Table S15 Statistics of sing-cell RNA sequencing data comparison results.**

| Sample | C-0 | E-1 | E-3 | E-7 | E-13 |
| --- | --- | --- | --- | --- | --- |
| Number of Reads | 480,740,914 | 339,817,367 | 407,634,479 | 445,169,149 | 420,655,527 |
| Clean Data (Gb) | 72.11 | 50.97 | 61.14 | 66.78 | 63.10 |
| Valid Barcodes | 95.30% | 61.10% | 95.70% | 87.20% | 90.00% |
| Q30 Bases in RNA Reads | 91.10% | 89.60% | 91.30% | 90.00% | 90.20% |
| Q30 Bases in UMI | 92.20% | 91.90% | 90.60% | 92.50% | 92.10% |
| Estimated Number of Cells | 3,484 | 4,407 | 5,370 | 5,399 | 3,024 |
| Fraction Reads in Cells | 47.50% | 47.70% | 46.00% | 52.20% | 57.20% |
| Mean Reads per Cell | 137,985 | 77,108 | 75,909 | 82,454 | 139,105 |
| Median Genes per cell | 211 | 363 | 352 | 310 | 346 |
| Total Genes Detected | 16,663 | 17,435 | 18,091 | 17,723 | 16,758 |
| Reads Mapped Confidently to Genome | 75.00% | 44.10% | 74.70% | 67.30% | 70.30% |
| Reads Mapped Confidently to Intronic Regions | 6.00% | 3.50% | 5.50% | 4.80% | 4.70% |
| Reads Mapped Confidently to Exonic Regions | 47.90% | 28.90% | 49.30% | 45.20% | 47.20% |
| Reads Mapped Confidently to Transcriptome | 43.90% | 26.20% | 45.00% | 41.20% | 43.40% |

**Table S16 Cell number and intracellular gene content of single-cell RNA sequencing data (21,001 cells).**

| Cluster | | Cells number | Median Genes per Cell | Median UMI Counts per Cell |
| --- | --- | --- | --- | --- |
| Connective Tissue | CT Ⅰ | 12,930 | 319 | 726.5 |
|  | CT Ⅱ | 1,496 | 1221 | 3275.5 |
| Muscle | | 2,490 | 350 | 660.5 |
| Nerve | | 2,725 | 626.5 | 1693 |
| Immune | | 914 | 280.5 | 675.5 |
| Endothelial | | 446 | 305.5 | 724.5 |

**Table S17 Marker gene information of different tissue and cell groups.**

| Cell type | Gene name | *P*-value | Function |
| --- | --- | --- | --- |
| CT Ⅰ | *cofilin* | 0.00 | Regulation of epithelial cell morphology and cytoskeleton also plays a role in embryonic cell division |
|  | *RPL8* | 0.00 | Ribosome protein gene, which plays a role in embryonic development of sea urchins |
|  | *ACTB* | 0.00 | Regulates actin |
|  | *Profilin* | 9.46E-263 | Cytoskeletal regulation |
|  | *ARHGDIA* | 7.86E-244 | Promote the recombination of actin skeleton and regulate cell shape |
|  | *beta-thymosin* | 9.28E-211 | Cytoskeletal organization that inhibits actin polymerization |
|  | *EIF5A* | 1.93E-193 | Neural development and differentiation of skeletal muscle stem cells play an important role |
|  | *ZFP36* | 6.47E-190 | Specific expression in the extraembryonic structure |
|  | *Eef1e1* | 1.43E-175 | Involved in translation quality control, also involved in cytoskeletal organization |
|  | *EGR1* | 4.64E-139 | Involved in inflammatory response, regulation of cell proliferation |
|  | *Lamins* | 1.87E-125 | Intermediate filamentous proteins that form the scaffold of the cell |
| Muscle | *Speg* | 0.00 | Striated muscle specific serine/threonine protein kinase |
|  | *UNC-89* | 0.00 | Muscle assembly protein |
|  | *Mp20* | 0.00 | Muscle specific protein |
|  | *SMTN* | 0.00 | Smooth muscle marker gene |
|  | *Muscle LIM Protein* | 0.00 | Structural maintenance of muscle cells |
|  | *Titin* | 0.00 | connecting |
|  | *ATP2A2* | 0.00 | ATP hydrolase mediates calcium ions from the cytoplasm to the sarcoplasmic reticulum |
|  | *SKI* | 0.00 | It plays a role in the terminal differentiation of skeletal muscle cells |
|  | *Mhc* | 0.00 | myosin heavy chain |
|  | *DLG5* | 1.55E-118 | Regulatory factors of Hippo signaling pathway |
| Nerve | *ADCY3* | 0.00 | Involved in the triggering of odor receptor signals |
|  | *PCSK2* | 0.00 | It is expressed in neurons and is related to neuroendocrine transformation |
|  | *CPLX1* | 0.00 | Positively regulates exocytosis of various vesicles |
|  | *ELAV* | 0.00 | RNA binding protein gene, necessary for nervous system development |
|  | *Syt1* | 0.00 | Calcium sensors involved in triggering the release of synaptic neurotransmitters |
|  | *Syt7* | 0.00 | Involved in secretion and Ca^2+^ dependent exocytosis of synaptic vesicles |
|  | *KCNK13* | 0.00 | Regulates the release of neurotransmitters |
|  | *Celsr2* | 0.00 | It plays an important role in cell/cell signaling during nervous system formation |
|  | *Ncan* | 0.00 | Regulates nerve cell adhesion and neurite growth |
|  | *CPE* | 0.00 | It acts as a prohormone processing enzyme in nerve cells |
|  | *Phm* | 0.00 | Catalyze C-terminal amidation of neuropeptides |
|  | *STMN1* | 0.00 | Necessary for axon formation during neurogenesis |
|  | *sox17a-a* | 2.78E-235 | It plays a downstream role in signal transduction in endoderm differentiation |
| Immune | *DMBT1* | 0.00 | It plays a role in mucosal defense, cellular immune defense and epithelial differentiation. |
|  | *Gp-340/SRCR* | 0.00 | Direct interaction with pathogenic microorganisms involved in innate immunity |
|  | *deleted in malignant brain tumors 1 protein-like isoform X2* | 0.00 | Same with *DMBT1* |
|  | *Apolipophorins* | 0.00 | involved in lipid transport |
|  | *FABP3* | 1.18E-178 | Participation in innate immunity |
|  | *Psap* | 3.88E-123 | Involved in the metabolism of macrophages |
|  | *Gnb2l1* | 4.49E-26 | Participate in the recruitment and assembly of signal molecules |
|  | *eIF4E* | 7.40E-07 | Restriction of protein synthesis |
| Endothelial | *MUC5AC* | 0.00 | Protects mucous membranes from infection and chemical damage |
|  | *FCGBP* | 0.00 | Participate in the maintenance of mucosal structure |
|  | *MFGE8* | 0.00 | Maintain epithelial homeostasis and promote mucosal healing |
|  | *PGRP-SC2* | 0.00 | Degrading bacterial peptidoglycan is involved in innate immunity |
|  | *Sema1a* | 5.18E-89 | Involved in growth cone guidance |
|  | *LRPAP1* | 2.30E-41 | Regulates lipoprotein ligand binding activity along the secretory pathway |
|  | *ADIPOR2* | 5.23E-20 | Involved in rebuilding blood vessel function |
|  | *Pc* | 9.44E-15 | Regulating gene expression involved in muscle tube development plays an important role in skeletal muscle growth and regeneration |
| CT Ⅱ | *Naca* | 0.00 | Regulating gene expression involved in muscle tube development plays an important role in skeletal muscle growth and regeneration |
|  | *Lrp6* | 0.00 | Cell surface co-receptor for Wnt/β-catenin signaling, essential for embryonic heart development |
|  | *MFAP4* | 0.00 | Extracellular matrix proteins belong to the fibrinogen associated protein superfamily |
|  | *ANGPTL1* | 0.00 | It plays an important role in vascular system development and maintenance |
|  | *VEGFR-3* | 0.00 | Promote the formation of endothelial system |
|  | *Tolloid* | 0.00 | It affects dorsal-ventral patterns and bone formation during development. |
|  | *Adamts6* | 0.00 | metallopeptidase |
|  | *Hyalin* | 0.00 | t acts as a substrate for cell adhesion during early development in echinoderms |
|  | *BP10* | 0.00 | Possibly involved in differentiation of ectodermal lineages and subsequent embryonic development |
|  | *TIMP1* | 0.00 | Metalloproteinases, as a growth factor, regulate cell differentiation, migration and cell death |
|  | *Fn1* | 0.00 | Adhesion proteins bind to cell surfaces and various compounds, including collagen and fibrin |
|  | *COL9A3* | 0.00 | Structural components of connective tissue, such as hyaline cartilage |
|  | *MMP2* | 0.00 | Degradation of type Ⅳ collagen, whose activity is mainly due to matrix metalloproteinase 2 tissue inhibitors |
|  | *FKBP14* | 0.00 | Speed up collagen synthesis |
|  | *Ppn* | 3.60E-110 | ssential extracellular matrix (ECM) protein that influences cell rearrangements. May act by modulating metalloproteinases action during organogenesis. |

**Table S18 Statistical table of cell numbers for each cell cluster at different regeneration stage.**

| Cluster | | E-1 | E-3 | E-7 | E-13 | C-0 |
| --- | --- | --- | --- | --- | --- | --- |
| Total | | 4,211 (100.00%) | 5,279 (100.00%) | 5,290 (100.00%) | 2,931 (100.00%) | 3,290 (100.00%) |
| Connective Tissue | CT Ⅰ | 2,753 (65.38%) | 3,169 (60.03%) | 3,248 (61.40%) | 1,910 (58.05%) | 1,850 (63.12%) |
|  | CT Ⅱ | 286 (6.79%) | 394 (7.46%) | 431 (8.15%) | 139 (2.31%) | 246 (8.39%) |
| Muscle | | 433 (10.28%) | 459 (8.69%) | 531 (10.04%) | 312 (10.64%) | 755 (22.95%) |
| Nerve | | 554 (13.16%) | 856 (16.22%) | 684 (12.93%) | 340 (11.60%) | 291 (8.84%) |
| Immune | | 126 (2.99%) | 252 (4.77%) | 255 (4.82%) | 117 (3.99%) | 164 (4.98%) |
| Endothelial | | 59 (1.4%) | 149 (2.82%) | 141 (2.67%) | 66 (2.25%) | 31 (0.94%) |

**Table S19 Marker gene information of each cell group in CT Ⅰ.**

| Cell type | Gene | *P* value | Function |
| --- | --- | --- | --- |
| MSC | *Gon-1* | 0.00 | Secreted metalloprotease required for distal tip cell (DTC) migration along the body wall basement membranes |
|  | *EGF3* | 0.00 | Forms the apical lamina, a component of the extracellular matrix |
|  | *Lrp6* | 1.16E-303 | Cell surface co-receptor for Wnt/β-catenin signaling, essential for embryonic heart development |
|  | *GPR126* | 1.44E-288 | This gene encodes a G protein-coupled receptor. Variations in this gene can affect stature. |
|  | *Pofut1* | 2.06E-287 | Plays a crucial role in NOTCH signaling. |
|  | *NOTCH1* | 1.34E-274 | Regulate cell-fate determination |
|  | *Fibrosurfin* | 9.72E-274 | fibrosurfins occurred between or near the collagen fibrils of the spinal ligament. |
|  | *SLC6A6* | 5.09E-268 | Sodium-dependent taurine and beta-alanine transporter. Chloride ions are necessary for optimal uptake. |
|  | *Gng7* | 4.72E-203 | Guanine nucleotide-binding proteins (G proteins) are involved as a modulator or transducer in various transmembrane signaling systems. |
|  | *ECM3* | 4.48E-114 | Extracellular matrix protein that may serve as substrate for the migratory primary mesenchyme cells (PMCs) |
|  | *PRDX6* | 2.71E-59 | Plays a role in cell protection against oxidative stress by detoxifying peroxides and in phospholipid homeostasis |
|  | *TRAF3* | 1.11E-48 | Regulates pathways leading to the activation of NF-kappa-B and MAP kinases, and plays a central role in the regulation of B-cell survival. |
|  | *RDX* | 1.35E-34 | Probably plays a crucial role in the binding of the barbed end of actin filaments to the plasma membrane. |
| Glial fibroblasts cells | *A2M* | 0.00 | Alpha macroglobulins are large glycoproteins which are present in the body fluids of both invertebrates and vertebrates. |
|  | *Nrx-IV/neurexin-4* | 0.00 | Septate junctions, which are the equivalent of vertebrate’s tight junctions |
|  | *COL2A1* | 0.00 | Collagen protein is a minor connective tissue component |
|  | *COL1A1* | 0.00 |  |
|  | *COL5A1* | 0.00 |  |
|  | *COL5A3* | 0.00 |  |
|  | *FSTL1* | 0.00 | Secreted glycoprotein that is involved in various physiological processes, such as regulation of the immune response |
|  | *l-2* | 0.00 | Involved in host defense at the body surface |
|  | *let-2* | 0.00 | Collagen type IV |
|  | *Sparcl1* | 0.00 | Secreted protein, acidic and rich in cysteine (SPARC) is a regulator of cell-matrix interaction |
|  | *Pard3* | 4.82E-194 | Adapter protein involved in asymmetrical cell division and cell polarization processes.Seems to play a central role in the formation of epithelial tight junctions |
| Glial effector cell | *His2Av* | 0.00 | Variant histone H2A which replaces conventional H2A in a subset of nucleosomes. |
|  | *Tmsb10* | 1.42E-283 | Plays an important role in the organization of the cytoskeleton. |
|  | *Arhgdia* | 1.41E-228 | Through the modulation of Rho proteins, may play a role in cell motility regulation. In glioma cells, inhibits cell migration and invasion by mediating the signals of SEMA5A and PLXNB3 that lead to inactivation of RAC1. |
|  | *PSMB3* | 5.04E-207 | Plays a key role in the maintenance of protein homeostasis |
|  | *EIF2S2* | 1.38E-186 | eIF-2 functions in the early steps of protein synthesis by forming a ternary complex with GTP and initiator tRNA. |
|  | *SET* | 2.43E-182 | Multitasking protein, involved in apoptosis, transcription, nucleosome assembly and histone chaperoning. |
|  | *ATPsynO* | 5.53E-180 | Mitochondrial membrane ATP synthase |
|  | *lmna* | 2.62E-179 | Provide a framework for the nuclear envelope and may also interact with chromatin. |
|  | *PPIL3* | 6.27E-159 | PPIases accelerate the folding of proteins. It catalyzes the cis-trans isomerization of proline imidic peptide bonds in oligopeptides. |
|  | *PHB* | 3.27E-157 | Protein with pleiotropic attributes mediated in a cell-compartment- and tissue-specific manner, which include the plasma membrane-associated cell signaling functions, mitochondrial chaperone, and transcriptional co-regulator of transcription factors in the nucleus |
|  | *COF1* | 2.09E-151 | Controls reversibly actin polymerization and depolymerization in a pH-sensitive manner. |
|  | *FSCN1* | 3.00E-143 | Actin-binding protein that contains 2 major actin binding sites |
| State sensing cells | *KRTAP5-8* | 0.00 | hair keratin intermediate filaments are embedded in an interfilamentous matrix, consisting of hair keratin-associated protein (KRTAP) |
|  | *HTR1F* | 0.00 | G-protein coupled receptor for 5-hydroxytryptamine (serotonin). Also functions as a receptor for various alkaloids and psychoactive substances |
|  | *SCOP2* | 0.00 | Visual pigments are the light-absorbing molecules that mediate vision. |
|  | *ZSWIM2* | 0.00 | E3 ubiquitin-protein ligase involved in the regulation of Fas-, DR3- and DR4-mediated apoptosis. |
|  | *Agrin* | 0.00 | plays a central role in the formation and the maintenance of the neuromuscular junction |
|  | *AHSA1* | 0.00 | Acts as a co-chaperone of HSP90AA1 |
|  | *Tamalin* | 0.00 | Plays a role in intracellular trafficking and contributes to the macromolecular organization |
|  | *ZFAND6* | 0.00 | Involved in regulation of TNF-alpha induced NF-kappa-B activation and apoptosis. |
|  | *CDC37* | 0.00 | Co-chaperone that binds to numerous kinases and promotes their interaction with the Hsp90 complex |
|  | *HSP110* | 0.00 | Belongs to HSP gene family, Participating in the stress response |
|  | *HSP70IV* | 0.00 |  |
|  | *Egr1-a* | 0.00 | EGR1 is important for numerous physiological processes including synaptic plasticity, wound repair, inflammation, and differentiation. |
|  | *Bag3* | 0.00 | Co-chaperone for HSP70 and HSC70 chaperone proteins. |
|  | *DRD3* | 9.48E-296 | Promotes cell proliferation. |
|  | *SLC6A9* | 9.94E-293 | May play a role in regulation of glycine levels in NMDA receptor-mediated neurotransmission. |
|  | *Ptprt* | 2.70E-197 | May be involved in both signal transduction and cellular adhesion in the CNS. |
|  | *rnf213a* | 2.37E-173 | Involved in the non-canonical Wnt signaling pathway in vascular development. Also involved in neuromuscular regulation |

**Table S20 Marker gene information of each cell group in CT Ⅱ.**

| Cell type | Gene | *P* value | Function |
| --- | --- | --- | --- |
| Cartilage-like Cells | *COL9A1* | 1.37E-69 | Structural component of hyaline cartilage |
|  | *TIMP2* | 5.09E-63 | Complexes with metalloproteinases (such as collagenases) and irreversibly inactivates them by binding to their catalytic zinc cofactor. |
|  | *RACK1* | 1.58E-54 | Scaffolding protein involved in the recruitment, assembly and/or regulation of a variety of signaling moleculeS. Rack1 oppositely regulates Wnt/β-catenin and Sonic hedgehog (Shh) signaling pathways in distinct developmental stages. |
|  | *Bmper* | 3.09E-45 | Inhibitor of bone morphogenetic protein (BMP) function, it may regulate BMP responsiveness of osteoblasts and chondrocytes. |
|  | *GM2A* | 9.84E-37 | Binds gangliosides and stimulates ganglioside GM2 degradation. It stimulates only the breakdown of ganglioside GM2 and glycolipid GA2 by beta-hexosaminidase A. |
|  | *COL2A1* | 3.67E-30 | Type II collagen is specific for cartilaginous tissues. It is essential for the normal embryonic development of the skeleton, for linear growth and for the ability of cartilage to resist compressive forces. |
|  | *COL1A1* | 1.92E-29 | Type I collagen is a member of group I collagen (fibrillar forming collagen). |
|  | *LvN1.2* | 1.75E-20 | LvN1.2 is an endoderm-specific gene |
|  | *Afdn* | 1.98E-17 | Belongs to an adhesion system, probably together with the E-cadherin-catenin system, which plays a role in the organization of homotypic, interneuronal and heterotypic cell-cell adherens junctions |
| Myogenic progenitor Cell | *MFAP4* | 1.48E-205 | Could be involved in calcium-dependent cell adhesion or intercellular interactions. May contribute to the elastic fiber assembly and/or maintenance |
|  | *AGRN* | 5.11E-124 | Plays a central role in the formation and the maintenance of the neuromuscular junction |
|  | *Lrp6* | 1.28E-117 | Cell surface co-receptor for Wnt/β-catenin signaling, essential for embryonic heart development |
|  | *SLC4A10* | 4.32E-117 | Sodium/bicarbonate cotransporter which plays an important role in regulating intracellular pH |
|  | *Chrnb1* | 1.02E-112 | After binding acetylcholine, the AChR responds by an extensive change in conformation that affects all subunits and leads to opening of an ion-conducting channel across the plasma membrane. |
|  | *ECM3* | 8.43E-110 | Extracellular matrix protein that may serve as substrate for the migratory primary mesenchyme cells (PMCs), the interaction possibly providing guidance information to migrating PMCs. |
|  | *Fcer2* | 6.73E-108 | Low-affinity receptor for immunoglobulin E (IgE) and CR2/CD21. Has essential roles in the regulation of IgE production and in the differentiation of B-cells (it is a B-cell-specific antigen). |
|  | *SLC4A2* | 8.81E-98 | Plasma membrane anion exchange protein of wide distribution. |
|  | *Naca* | 1.55E-84 | Cardiac- and muscle-specific transcription factor. May act to regulate the expression of genes involved in the development of myotubes. |
|  | *ANGPT4* | 9.93E-56 | Binds to TEK/TIE2, modulating ANGPT1 signaling. Promotes endothelial cell survival, migration and angiogenesis. |
|  | *COL13A1* | 2.97E-53 | Involved in cell-matrix and cell-cell adhesion interactions that are required for normal development. |
|  | *COL4A5* | 5.08E-42 | Type Ⅳ collagen |
| Functional endothelial-like Cell | *PstI/PR1* | 6.59E-213 | degrading protease |
|  | *CDPs* | 9.87E-198 | Cuticle degrading proteases |
|  | *Klkb1* | 5.99E-103 | Encodes a glycoprotein that participates in the surface-dependent activation of blood coagulation, fibrinolysis, kinin generation and inflammation |
|  | *proteinase T* | 4.49E-94 | Serine proteinase. |
|  | *CPA2* | 5.00E-90 | a zinc-containing metalloprotease that removes the amino acid residue from the C-terminal of a peptide chain. |
|  | *SYT1* | 1.42E-32 | Calcium sensor that participates in triggering neurotransmitter release at the synapse |
|  | *CPD* | 6.45E-17 | Carboxypeptidase D |
|  | *Sqstm1* | 1.75E-12 | Autophagy receptor required for selective macroautophagy (aggrephagy). |
|  | *CDK5R1* | 1.8E-11 | p35 is a neuron specific activator of CDK5. The complex p35/CDK5 is required for neurite outgrowth and cortical lamination. |
| Epithelial Cell | *TLL1* | 8.83E-143 | Predominant protease, which in the development, influences dorsal-ventral patterning and skeletogenesis. |
|  | *ADAM9* | 3.13E-130 | Cleaves and releases a number of molecules with important roles in angiogenesis |
|  | *BP10* | 4.38E-128 | Possibly involved in differentiation of ectodermal lineages and subsequent embryonic development |
|  | *SpAN* | 1.11E-123 | Asymmetrically along the animal-vegetal axis of the blastula. |
|  | *MFGE8* | 2.53E-84 | Plays an important role in the maintenance of intestinal epithelial homeostasis and the promotion of mucosal healing. |
|  | *wbfib4* | 1.02E-65 | Zinc metalloproteinase |
|  | *Fcer2* | 5.26E-65 | Low-affinity receptor for immunoglobulin E (IgE) and CR2/CD21. Has essential roles in the regulation of IgE production and in the differentiation of B-cells (it is a B-cell-specific antigen). |
|  | *Ppn* | 5.45E-37 | Essential extracellular matrix (ECM) protein that influences cell rearrangements. May act by modulating metalloproteinases action during organogenesis. |
|  | *Mzb1* | 4.03E-17 | Associates with immunoglobulin M (IgM) heavy and light chains and promotes IgM assembly and secretion. |
|  | *RASEF* | 4.68E-17 | Acts as a dynein adapter protein that activates dynein-mediated transport and dynein-dynactin motility on microtubules |
|  | *BAD* | 1.61E-14 | Promotes cell death. Successfully competes for the binding to Bcl-X(L), Bcl-2 and Bcl-W, thereby affecting the level of heterodimerization of these proteins with BAX. |
|  | *APLP1* | 2.45E-05 | Couples to JIP signal transduction through C-terminal binding. May interact with cellular G-protein signaling pathways. |
| Juxtaligamental-like cell | *CLEC3B* | 2.69E-222 | May be involved in the packaging of molecules destined for exocytosis. |
|  | *NEUROD1* | 1.06E-193 | Associates with the p300/CBP transcription coactivator complex to stimulate transcription of the secretin gene as well as the gene encoding the cyclin-dependent kinase inhibitor CDKN1A. |
|  | *ITGBL1* | 2.72E-138 | This gene encodes a beta integrin-related protein that is a member of the EGF-like protein family. |
|  | *Rxfp2* | 1.97E-104 | Receptor for relaxin. The activity of this receptor is mediated by G proteins leading to stimulation of adenylate cyclase and an increase of cAMP. |
|  | *SibA* | 2.64E-100 | Implicated in cellular adhesion to substrate or phagocytic particles. |
|  | *Foxb1* | 5.67E-77 | Transcription factor. May be involved in the early anteroposterior patterning of the neuroectoderm. |
|  | *PCSK2* | 3.72E-73 | Interacts with a neuroendocrine secretory protein in the ER, exits the ER and sorts to secretory granules |
|  | *Insm1a* | 6.15E-62 | May act as a transcriptional regulator. May play a role in neurogenesis and neuroendocrine cell differentiation during embryonic development |
|  | *Syt12* | 2.84E-52 | Synaptic vesicle phosphoprotein that enhances spontaneous neurotransmitter release but does not effect induced neurotransmitter release |
|  | *NRSN1* | 6.87E-52 | Play an important role in neural organelle transport, and in transduction of nerve signals or in nerve growth. May play a role in neurite extension. |
|  | *TRPC4* | 2.76E-48 | Forms a receptor-activated non-selective calcium permeant cation channel. Acts as a cell-cell contact-dependent endothelial calcium entry channel. |
|  | *App* | 9.50E-36 | Functions as a cell surface receptor and performs physiological functions on the surface of neurons relevant to neurite growth, neuronal adhesion and axonogenesis. |
|  | *FEZ2* | 1.89E-29 | Involved in axonal outgrowth and fasciculation. |
|  | *Gyc76C* | 5.99E-90 | Plays a role in Sema-1a-mediated axon repulsion which is required for the correct establishment of neuromuscular connectivity |
|  | *Syt7* | 1.52E-26 | Ca^2+^ sensor involved in Ca^2+^-dependent exocytosis of secretory and synaptic vesicles through Ca2+ and phospholipid binding to the C2 domain |
|  | *Shtn1* | 1.84E-23 | Involved in the generation of internal asymmetric signals required for neuronal polarization and neurite outgrowth |
|  | *Cnga4* | 2.85E-23 | Causes the opening of cation-selective cyclic nucleotide-gated (CNG) channels and depolarization of the neuron (olfactory sensory neurons, OSNs). |

**Table S21 Expression of core markers (between axolotl and ophiuroid) in *Ophiura sarsii vadicola***

| Gene ID | D1 | D3 | D7 | D13 | D0 | Annotation |
| --- | --- | --- | --- | --- | --- | --- |
| evm.TU.CTG.1291.12 | 13.36 | 13.61 | 14.73 | 21.00 | 6.21 | PREDICTED: 60S acidic ribosomal protein P1-like [Saccoglossus kowalevskii] |
| evm.TU.CTG.1292.19 | 9.24 | 8.45 | 7.73 | 11.06 | 3.52 | PREDICTED: 40S ribosomal protein S20 [Strongylocentrotus purpuratus] |
| evm.TU.CTG.140.7 | 7.40 | 7.56 | 6.51 | 9.77 | 2.72 | PREDICTED: 60S acidic ribosomal protein P2 [Coturnix japonica] |
| evm.TU.CTG.1735.78 | 8.29 | 8.30 | 6.94 | 10.91 | 3.30 | AAEL015006-PA [Aedes aegypti] |
| evm.TU.CTG.1916.17 | 10.89 | 9.09 | 9.83 | 13.03 | 3.90 | PREDICTED: 60S ribosomal protein L30 isoform X1 [Strongylocentrotus purpuratus] |
| evm.TU.CTG.1916.33 | 10.68 | 8.83 | 8.03 | 11.52 | 3.79 | PREDICTED: ubiquitin-40S ribosomal protein S27a [Lingula anatina] |
| evm.TU.CTG.1969.9 | 11.49 | 9.97 | 10.85 | 16.37 | 5.51 | PREDICTED: 60S ribosomal protein L44 [Priapulus caudatus] |
| evm.TU.CTG.2025.15 | 7.78 | 7.26 | 6.09 | 9.78 | 2.48 | ribosomal protein S14 [Strongylocentrotus purpuratus] |
| evm.TU.CTG.2289.5 | 14.38 | 11.65 | 12.13 | 19.24 | 5.83 | PREDICTED: 60S ribosomal protein L7a-like [Saccoglossus kowalevskii] |
| evm.TU.CTG.3206.19 | 16.11 | 14.83 | 14.75 | 24.49 | 7.74 | 40S ribosomal protein S23 [Sycon ciliatum] |
| evm.TU.CTG.3737.14 | 10.96 | 10.06 | 9.37 | 14.18 | 4.37 | PREDICTED: 60S ribosomal protein L35-like [Saccoglossus kowalevskii] |
| evm.TU.CTG.3762.7 | 10.72 | 11.04 | 10.44 | 13.97 | 4.89 | 40S ribosomal protein s27 [Ictalurus furcatus] |
| evm.TU.CTG.684.7 | 9.52 | 8.65 | 7.67 | 13.59 | 4.08 | PREDICTED: 60S ribosomal protein L32 [Strongylocentrotus purpuratus] |

**Table S22 The sequence information for probes used in the whole mount in situ hybridization experiment.**

| Gene name | Sequence（5’-3’） |
| --- | --- |
|  |  |
| *Lrp6* | TTGAGTCAACTTCCGTGCAAGCATCGAGGAAATCTATATTTTCCGCTCACATGAGAGCCAAAGTATTATAATAGGTGCCGAGTCATCAAACCTAATTTACCTTAAGGACAATCAAAGCCATGGTCTTGACCTTATTTTCTTGAATTATTTTCAGGCATGATATTTGAGAGTGAATCATTAATGCCTTATACAGTCGGTGGAGATAATTTTAGTTCTCGGCTATATATTTGATATATGTTATAAACTCACTTAATGTCACTCGGCAATGTTTGTTTTGCCTGAGATGAAAGTAGATATTTGGCAGTACTGCCAAATCAGCTCAAATCAGTACGTATAGCTCAGACGTATAATTGACGTAAAATGTTAAACATTTGGTGACTATCGTATAAGTCTTCCGCGCAAGTCACTTATACGCATGAGCCACACGTACTAGTTTTGCAGTTAATCCTTATAATCTACGTGATCAAAGCGCTGATGACATCCGTGTACCACACATGCAGTATGCTTAAATGAAGCGATATCATTTTGATCATGTTTCTGATGATTGAGTCCGTTTTGAGAAAGTCGCTATATCATATCAAGTATGATTTATAACATAATACAATTATCTTGCCAATGTAATGCAAGCAAATATCAAAACCAACGTTTATGACTTCATACAATACGATTTTGGTTGAATCTTGATCAGATGGTTTTCAAGGGAAAAGGGAAATGAGACTGAAACAAAAAGCGACTGAAACAAAAAGCATGTAACTTAAACGTCAGATATTGTTTTCATTTAAAATTCATTAAGCAATTTTCATTTAGGAGACATTGCGAAATCAGTTTGCTGCACTCGTGCGTAAAAGTGACTTTTACCCTAGACATTATTAAACGATATTTCTATAGTCTATTTGCAGCACATAACGCGCGCGCCTACTCGAGCCGCTGGCGTATATTCACGAGCGTTGAAAGGACGGAAG |
| *Fibrosurfin* | TTTGAGTACGGCCCAAAGTGATACACGCCAGCTTATTGCACAGCACTTATTATTCACTGAGGCCCGTTTAGTACTGATTTCTTCATATATAATTATACATATTTCTATCTAAATATCTTTGTTTCTTAATCTAATATTCACATTTTACCCCCATACCAATAACCAATACACATAGGTATTATCATAATTGAATAAAATTGAGTTGATAACATGAGTAAAAATATATTTATAATGTGATGTCAACAATTTTGTACATCAATCATTTTTATCCTTACAAATAGTTAGTAATTAAAACAAAAAATCAAAACAAAGCTTTATCCATGAAGTGGATACATTAAAGAAACTTCATATAGTGAAATTAACCAATTGTAAAATTATTTAATATTTGCTTGCAAAATTGTCTACTCCCATCAGTAACTGGTCAATTATTTTGTGTCTTTAATTTAACTTTTCTGTCATATCGCCTTTGTTTATGTAATCATTCATTATATAACACTATATATGATGTATAACACTACGGTAACATTAATATAAAAATATATTTCAATTTTCTTATAAAAATAAGTCAAACCCCCAAAAGCTTTAATTTGAGTGATTCCATATATTTCCAATGAAACAAGCTTCTAAGTGTCTAATGAACTGTGTATTATTTTAATATTTGGATCAATGCTGCTTCAATACATTACAAGATTGATCCTTAAAACAGCATCGATTACAGCCATTATTTACAGTTTTTGTYGAGTATATTGAGTATTGAGTTTTTGTCTATAAAACATCATTGTGATTCTGGTTCGCTGTATGTATCCAAGTGAATACCCCCCTCATGGGGTTTAATAGGTGTCTGACGTTACCTGGTCACTGTTTTCGTGAAGCA |
