# Supplementary material for: Chromosome-level genome assembly and single-cell analysis unveil molecular mechanisms of arm regeneration in the ophiuroid Ophiura sarsii vadicola
Source: Genome Biol. 2025 Mar 31;26:82. doi: 10.1186/s13059-025-03542-5 (PMC11959729; doi:10.1186/s13059-025-03542-5)
Supplement: Supplementary file 1 — Additional file 1: Figures S1–S12 [file 13059_2025_3542_MOESM1_ESM.docx]

**Chromosome-level genome assembly and single-cell analysis unveil molecular mechanisms of arm regeneration in the ophiuroid *Ophiura sarsii vadicola***

Supplementary figures

Fig. S1 k-mer distribution of *Ophiura sarsii vadicola* using Illumina reads.

Fig. S2 HI-C interaction heatmap of the genome assembly. Axis shows the chromosome ID.

Fig. S3 The gene function annotation information of *Ophiura sarsii vadicola.*

Fig. S4 Annotation quality comparison of protein-coding genes for *Ophiura sarsii vadicola* and three echinoderm genomes used for annotation.

Fig. S5 Venn diagram of shared gene families between three ophiuroid genomes.

Fig. S6 The morphology observation of regeneration at the tips of arms for *Ophiura sarsii vadicola* cultured in laboratory.

Fig. S7 The cluster and heatmap of correlations between transcriptome samples of the *Ophiura sarsii vadicola.*

Fig. S8 Distribution of basic cell information of each sample before and after filtration. (first line: before filtration; second line: after filtration).

Fig. S9 Preliminary clustering results of single cell RNA sequencing data (A) and the different resolution of clustering strategy (B).

Fig. S10 The Whole Mount In Situ Hybridization of regeneration at the tips of arms for *Ophiura sarsii vadicola* cultured in the laboratory.

Fig. S11 Pseudo-timing analysis of CT Ⅱ cells during regeneration using Slingshot.

Fig. S12 Cross-species cell type similarity analysis for the axolotl and brittle star.

Fig. S1 k-mer distribution of *Ophiura sarsii vadicola* using Illumina reads.

Fig. S2 HI-C interaction heatmap of the genome assembly. Axis shows the chromosome ID.

Fig. S3 The gene function annotation information of *Ophiura sarsii vadicola*.

Fig. S4 Annotation quality comparison of protein-coding genes for *Ophiura sarsii vadicola* and three echinoderm genomes used for annotation.

#Aja: *Apostichopus japonicus*; Apl: *Acanthaster planci*; Spu: *Strogylocentrotus purpuratus*

Fig. S5 Venn diagram of shared gene families between three ophiuroid genomes.

Fig. S6 The morphology observation of regeneration at the tips of arms for *Ophiura sarsii vadicola* cultured in laboratory.

A and A’, Day 1 after amputation; B and B’, Day 3 after amputation; C and C’, Day 7 after amputation; D and D’, Day 13 after amputation; E and E’, Day 0 after amputation.

Fig. S7 The cluster and heatmap of correlations between transcriptome samples of the *Ophiura sarsii vadicola*.

Fig. S8 Distribution of basic cell information of each sample before and after filtration. (first line: before filtration; second line: after filtration).

Fig. S9 Preliminary clustering results of single cell RNA sequencing data (A) and the different resolution of clustering strategy (B). Groups in A: C-0, control (0 day); E-1, first day after amputation (day 1); E-3, third day after amputation (day 3) ; E-7, seventh day after amputation; E-13, thirteenth day after amputation. B: circles in each line, suggested cluster group.

Fig. S10 The Whole Mount In Situ Hybridization of regeneration at the tips of arms for *Ophiura sarsii vadicola* cultured in the laboratory.

A-D is the hybridization result of *fibrosurfin*, and E-H is the hybridization result of *LRP6*. A, E (day 1) and B, F (day 3) in the wound healing stage, and C, G (day 13) and D, H (day 0) in early regenerative phase. Arrows: the location of key gene. Scale bar, 2 mm.

Fig. S11 Pseudo-timing analysis of CT Ⅱ cells during regeneration using Slingshot.

Fig. S12 Cross-species cell type similarity analysis for the axolotl and brittle star.
